# Unveiling the mechanism of action of acylated temporin L analogues against multidrug-resistant *Candida albicans*

**DOI:** 10.1080/14756366.2022.2134359

**Published:** 2022-10-28

**Authors:** Rosa Bellavita, Falanga Annarita, Francesco Merlino, Gabriella D’Auria, Nicola Molfetta, Anella Saviano, Francesco Maione, Umberto Galdiero, Maria Rosaria Catania, Galdiero Stefania, Paolo Grieco, Emanuela Roscetto, Lucia Falcigno, Buommino Elisabetta

**Affiliations:** a Department of Pharmacy, University of Naples “Federico II”, Naples, Italy; b Department of Agricultural Science, University of Naples “Federico II”, Portici, Italy; c Department of Molecular Medicine and Medical Biotechnology, University of Naples Federico II, Naples, Italy

**Keywords:** Acylated peptides, temporins, *Candida albicans*, membrane interaction

## Abstract

The increasing resistance of fungi to conventional antifungal drugs has prompted worldwide the search for new compounds. In this work, we investigated the antifungal properties of acylated Temporin L derivatives, **Pent-1B** and **Dec-1B**, against *Candida albicans*, including the multidrug-resistant strains. Acylated peptides resulted to be active both on reference and clinical strains with MIC values ranging from 6.5 to 26 µM, and they did not show cytotoxicity on human keratinocytes. In addition, we also observed a synergistic or additive effect with voriconazole for peptides **Dec-1B** and **Pent-1B** through the checkerboard assay on voriconazole-resistant *Candida* strains. Moreover, fluorescence-based assays, NMR spectroscopy, and confocal microscopy elucidated a potential membrane-active mechanism, consisting of an initial electrostatic interaction of acylated peptides with fungal membrane, followed by aggregation and insertion into the lipid bilayer and causing membrane perturbation probably through a carpeting effect.

## Graphical abstract

**Figure:**
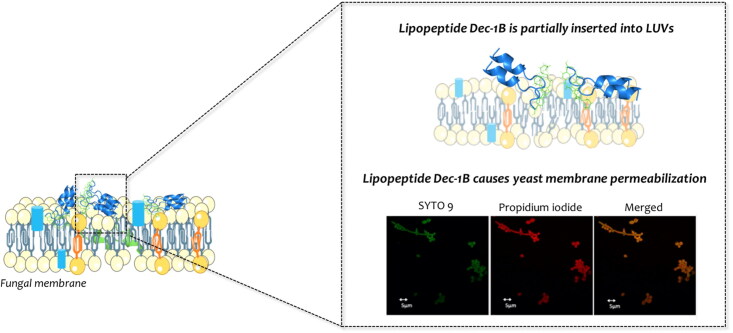

## Introduction

Fungal diseases affect billions of individuals and represent a worldwide threat to human health. The significant increase of fungal infections is likely due to the increased use of immunosuppressive therapies in cancer or transplantation, as well as medical devices and the indiscriminate consumption of antibiotics. Antifungal treatments are essential for reducing comorbidities and mortalities, but resistance-related issues have prompted the search for more effective alternatives with reduced toxicity, improved pharmacodynamics and pharmacokinetics, and increased specificity^1^. Candidiasis is the most notable infection caused by fungi of the *Candida* genus and represents a major concern for public health^2^. Species of *Candida* are the leading pathogens causing around 400,000 systemic fungal diseases worldwide^3^ and are considered the fourth most common cause of nosocomial blood infections, even overcoming Gram-negative pathogens^4^^,^^5^. Although more than one hundred *Candida* species exist, only a few are implicated in clinical infections. Of these, *C. albicans* is certainly the most isolated species from clinical specimens and it is generally found in 90–100% of mucosal isolates and 50–70% of blood infection isolates^6^. *Candida* spp infections exhibit different clinical manifestations, from superficial mucocutaneous candidiasis affecting mucosal, nail and skin surfaces, to systemic infections affecting organs^7^. These infections are commonly seen in immunocompromised patients or in those conditions in which yeast overgrowth is favored^7^. *Candida* species have developed mechanisms of resistance towards antifungal drugs commonly used for the treatment of systemic or topical infections^8^^,^^9^. Among them, azoles (e.g. fluconazole, voriconazole), polyenes (e.g. amphotericin B) and echinocandins (e.g. caspofungin) are the most efficacious as they specifically interfere with the ergosterol or cell wall biosynthesis, which are not present in mammalian cells^8^. For example, activities of the azoles are directed against lanosterol 14-α-demethylase (Erg11p), which is encoded by the ERG11 gene, involved in the ergosterol biosynthesis. Among resistant clinical isolates of *C. albicans*, ERG11 over-expression is frequently observed^10^. The numerous toxic effects, including liver and kidney dysfunctions, local phlebitis, haemolysis and fever^11^^,^^12^ caused by these antifungal drugs prompted the focus of recent research on new agents with improved safety profile and broad-spectrum antimicrobial activity for use alone and/or in synergy with antifungals.

Emerging drug candidates attracting the attention of researchers are antimicrobial peptides (AMPs), host defence peptides that play an essential role in the innate immune response^12^. AMPs, mostly isolated from living organisms, are typically featured by a net positive charge at physiological pH due to the presence of basic residues (e.g. Arg and Lys) while the hydrophobic residues determine their amphipathic conformation. Both the cationic nature and the secondary structure facilitate the interaction with the negatively charged membrane of microorganisms^12^^,^^13^, leading to insertion, destabilisation and disruption of the cell membrane, although they can also act on intracellular targets^14^^,^^15^. Since the cell membrane is the primary target of AMPs, microorganisms are less likely to develop a resistance mechanism, making AMPs promising drug candidates^16^.

Temporins are AMPs isolated from the skin of the frog *Rana temporaria*, and exhibit strong antimicrobial, anti-*Candida* and antiviral activities^17–19^. Temporin L (TL) is the most active isoform on both Gram-negative and Gram-positive bacteria and yeasts, but it has high haemolytic activity^18^^,^^20^. Previous structure-activity relationship studies (SARs) carried out by our research group led to the discovery of synthetic peptides derived from Temporin L possessing a strong ability to kill both Gram-positive and Gram-negative bacteria, and yeasts as *Candida* spp^21^. Interestingly, some of them have also demonstrated scarce or absent haemolytic activity at their antimicrobial concentrations^21^^,^^22^. Our group has deeply worked on a promising molecule derived from Temporin L, [Pro^3^DLeu^9^DLys^10^] TL (namely **1B**, sequence: Phe-Val-Pro-Trp-Phe-Ser-Lys-Phe-dLeu-dLys-Arg-Ile-Leu-CONH_2_), that has proved to be an efficient antimicrobial agent, also devoid of cytolytic effects *in vitro*^23^. Interestingly, peptide **1B** has demonstrated the ability to inhibit the growth of methicillin-resistant *Staphylococcus pseudintermedius* (MRSP), and *Malassezia pachydermatis* and the prolonged application of this peptide did not cause *M. pachydermatis* drug-resistance and increased the susceptibility of MRSP to oxacillin^24^. In addition, **1B** has shown anti-inflammatory activity *in vivo* in response to zymosan-induce peritonitis^25^. This result is worthy of note since it supports a possible application of **1B** as an anti-inflammatory and antimicrobial agent. These results have also been described in a patent which has granted on 7 May 2021 (Number: 102019000009489).

Approaches to reduce or eliminate the toxicity of conventional antifungal drugs and enhance their activity may include the use of drug delivery systems or novel strategies that likely improve the interaction with the target membranes^26–28^.

To improve the antimicrobial profile of peptide **1B**, we have previously applied a lipidation strategy^29^ consisting of the addition of fatty acids in *N-* and *C-*terminus of the peptide, and discovered the analogue **Pent-1B**, bearing a pentyl chain in *para* position of Phe^1^ (peptide C in Bellavita et al.^29^). This compound has shown a good activity against carbapenem resistant *Klebsiella pneumoniae* and a low cytotoxicity against both human erythrocytes and keratinocytes^29^^,^^30^.

Through detailed biophysical studies focussed on the mode of action of **Pent-1B** using bacterial biomembrane models, we demonstrated that **Pent-1B** has a low ability to aggregate in aqueous solution, while it has a significant ability to oligomerize in membrane mimetic environments. This causes an alteration of the membrane fluidity and, by creating pores in both Gram-positive and Gram-negative membranes, eventually leads to cell death^29^.

Based on the results until here obtained, herein, we were prompted to investigate the antifungal properties of **Pent-1B** against reference and clinical strains of *C. albicans*, and with the further aim to increase the affinity and interaction with fungal membrane, we designed and synthesised the peptide **Dec-1B** bearing a longer lipid tail of 10-carbon atoms. The antifungal properties of the peptide **Dec-1B** were investigated against *C. albicans,* while its cytotoxicity was assessed on human keratinocytes. The effect of **Pent-1B** and **Dec-1B** in combination with antifungal drug voriconazole was evaluated on voriconazole-resistant *C. albicans* strains. Moreover, the mechanism of action on fungal membrane models of peptides **Pent-1B** and **Dec-1B** was explored by fluorescence assays, including Thioflavin T and leakage assays, tryptophan quenching experiments, nuclear magnetic resonance (NMR) spectroscopy and confocal microscopy.

## Materials and methods

### Materials

The conventional Fmoc-amino acids and coupling reagents *N,N,N’,N’*-tetramethyl-O-(1H-benzotriazol-1-yl) uranium hexafluorophosphate (HBTU) and 1-hydroxybenzotriazole (HOBt), were commercially obtained by GL Biochem Ltd acquired from GL Biochem Ltd (Shanghai, China). The unconventional amino acid Boc-l-Phe(4-NO_2_)-OH, Rink amide resin (loading substitution of 0.70 mmol/g), *N,N-*diisopropylethylamine (DIEA), piperidine, triisopropylsilane (TIS) and trifluoroacetic acid (TFA) were purchased from Iris-Biotech GMBH. Tin(II) chloride anhydrous, valeric and decanoic acids were acquired from Sigma Aldrich-Merck. Moreover, peptide synthesis solvents, such as *N,N*-dimethylformamide (DMF), water and acetonitrile for HPLC, were reagent grade acquired from commercial sources (Sigma Aldrich-Merck and VWR) and used without further purification.

Phospholipids: 1-Palmitoyl-2-oleoyl-sn-glycero-3-phosphocholine (DOPC), L-α-phosphatidylethanolamine (PE), L-phosphatidylglycerol (PG), phosphatidylinositol (PI), 1-palmitoyl-2–(6,7-dibromo)stearoyl-sn-glycero-3-phosphocholine (6,7 Br-PC), 1-palmitoyl-2–(9,10-dibromo)stearoyl-sn-glycero-3-phosphocholine (9,10 Br-PC), and 1-palmitoyl-2–(11,12-dibromo)stearoyl-sn-glycero-3-phosphocholine (11,12 Br-PC) were purchased from Avanti Polar Lipids, Inc. (Alabaster, AL, USA). Ergosterol, 1,1,1,3,3,3-Hexafluoro-2-propanol, Nile Red (NR) and Thioflavin T were acquired from Sigma Aldrich-Merck. 8-aminonaphtalene-1,3,6-trisulfonic acid, disodium salt (ANTS) and p-xylene-bis-pyridinium bromide (DPX) were purchased from Molecular Probes. Deuterium oxide (D_2_O) of 99.9% isotopic purity and dimethyl sulfoxide (DMSO-d_6_) of 99.9% isotopic purity were purchased from Sigma Aldrich (Milan, Italy). Dodecylphosphorylcholine-d_38_ (DPC-d_38_) of 98% isotopic purity and 16-doxylstearic acid were purchased from Merck Life Science S.r.l. (Milan, Italy). Sodium-3-(trimethylsilyl) propionate 2,2,3,3-d4 (TSP) was from Cambridge Isotope Laboratories (CIL), Inc. (Andover, MA, USA). Antibiotics were purchased from Sigma-Aldrich (Milan, Italy).

### Candida *strains and culture conditions*

The yeast strains evaluated in this study included *C. albicans* ATCC 90028 (reference strain) and *C. albicans* ATCC 10231 (azole-resistant strain) obtained from the American Type Culture Collection (Rockville, MD), and six azole-resistant clinical isolates (*Ca*1*-Ca*6) belonging to an anonymous collection of fungal strains established at the Department of Molecular Medicine and Medical Biotechnology, University of Naples Federico II (Table 1). Identification was performed by subcultures on chromogenic agar (Chromid agar) (Becton Dickinson) and confirmed by MS MALDI-TOF (Bruker). Susceptibility to amphotericin B, anidulafungin, micafungin, caspofungin, posaconazole, voriconazole, itraconazole, and fluconazole was assessed using the Sensititre Yeast One colorimetric microdilution method (Thermofisher). The results were interpreted according to the MIC breakpoints reported by Clinical and Laboratory Standards Institute (CLSI)^31^ with the exception of the MICs of itraconazole, posaconazole and amphotericin B, which were interpreted according to European Committee on Antimicrobial Susceptibility Testing (Eucast)^32^. All strains were stored as 15% (*v/v*) glycerol stocks at −80 °C. Prior to each experiment, cells were subcultured from the stocks onto Sabouraud dextrose agar (SDA) (Becton Dickinson) at 37 °C for 48 h.

**Table 1.** Antimycotic-susceptibility profiles of clinical *C. albicans* used in our study.

*Candida albican*s clinical strains	MIC values (μg/mL) of antifungal agent*^a^*							
	AND	MF	CAS	PZ	VOR	IZ	FZ	AB
*Ca*1	1 (R)	4 (R)	2 (R)	0.25 (R)	0.5 (I)	0.25 (R)	64 (R)	0.5 (S)
*Ca*2	0.015 (S)	0.008 (S)	0.015 (S)	1 (R)	0.5 (I)	0.5 (R)	64 (R)	0.12 (S)
*Ca*3	0.03 (S)	0.008 (S)	0.06 (S)	8 (R)	>8 (R)	16 (R)	128 (R)	0.5 (S)
*Ca*4	0.06 (S)	0.125 (S)	0.06 (S)	1 (R)	8 (R)	16 (R)	128 (R)	0.5 (S)
*Ca*5	0.12 (S)	0.5 (I)	0.12 (S)	0.125 (R)	0.015 (S)	0.12 (R)	0.25 (S)	2 (R)
*Ca*6	1 (R)	1 (R)	0.5 (I)	0.125 (R)	0.03 (S)	0.12 (R)	1 (S)	0.5 (S)

*^a^*AND: anidulafungin; MF: micafungin; CAS: caspofungin; PZ: posaconazole^1^; VOR: voriconazole; IZ: itraconazole; FZ: fluconazole; AB: amphotericin B^1^, R, Resistant; S, Susceptible, I, intermediate. MIC values interpretated with clinical breakpoint EUCAST (version 10.0).

### Acylated peptide synthesis

The acyl-peptides **Pent-1B** and **Dec-1B** were synthesised by using ultrasounds-assisted solid-phase peptide synthesis (US-SPPS)^33^ and by employing the Rink amide resin as solid support^29^. In details, peptide assembly was obtained by performing repeated cycles of (i) Fmoc deprotections, by treatments with 20% of piperidine in DMF solution [0.5 + 1 min under ultrasonic irradiation), and (ii) coupling reactions, by treatment with a solution of Fmoc-AA(3 eq), HBTU (3 eq), HOBt (3 eq) and DIEA (6 eq) in DMF, and exposing the reagent mixture to the ultrasonic irradiation for 5 min. For the construction of lipidic tails, the amino acid Boc-l-Phe(4-NO_2_)-OH was used and coupled as the last residue to the peptide sequence. Therefore, prior conjugation to lipidic tails, the nitro group was reduced to primary amine by treatment with SnCl_2_ 1 M solution in DMF, at rt for 16 h. Then, valeric or decanoic acid (3 eq), together with HBTU (3 eq), HOBt (3 eq) and DIEA (6 eq) in DMF/DCM (1:1, *v/v*), were added to the resin-bound peptides **Pent-1B** and **Dec-1B**, respectively, and reactor was agitated on an automated shaker for 2 h. Once the lipidic tail coupling was ascertained by LC-MS analysis, peptides were released from the resin and all protecting groups were simultaneously cleaved by treatment with a cleavage cocktail of TFA/TIS/H_2_O (95:2.5:2.5, *v/v/v*), for 3 h under stirring. Finally, lipidated peptides were purified by preparative RP-HPLC equipped with a Phenomenex Kinetex column (C18, 5 µm, 100 Å, 150 × 4.6 mm) and by using a linear gradient of MeCN (0.1% TFA) in water (0.1% TFA) from 10 to 90% over 30 min, and the correct molecular masses were confirmed through electrospray ionisation mass spectrometry (ESI-MS) analyses (Table S1).

### Fluorescein-labelling of peptide dec-1B

The peptide **Dec-1B-K(Fam)** was labelled to 5(6)-carboxyfluorescein (Fam) on side chain of an additional Lys placed in *C*-terminus. Thus, Fmoc-l-Lys(Mtt)-OH was introduced during peptide elongation, and, after the construction of the target sequence, as above described, the fluorescein-labelling proceeded as follows. The Mtt group was selectively removed by treating the resin-bound peptide sequence with a cocktail of TFA/TIS/DCM (1:5:94, *v/v/v*), for 25 min at rt.^16^ The procedure was repeated seven times, and the complete deprotection was ascertained by the Kaiser test. The 5(6)-carboxyfluorescein (2 eq) was coupled by using COMU (2 eq), Oxyma (2 eq) and DIPEA (4 eq), and reagent mixture was allowed to stir for 25 min. Then, the resin was washed with DMF (× 3) and the coupling was repeated for an additional 25 min. The fluorescein labelling was ascertained by the LC-MS analysis. Finally, the peptide was cleaved from the resin and purified by preparative RP-HPLC equipped with a Phenomenex Kinetex column (C18, 5 µm, 100 Å, 150 × 4.6 mm) and a linear gradient of MeCN (0.1% TFA) in water (0.1% TFA) from 10 to 90% over 30 min, and the correct molecular mass was confirmed by ESI-MS analysis (Table S1).

### Determination of the minimum inhibitory concentration (MIC) and the minimum fungicidal concentration (MFC)

The antifungal activity of compounds was determined using a standardised broth microdilution method (Clinical and Laboratory Standards Institute (CLSI) document M27-A4) ^34^. Briefly, cell suspension was adjusted to 3 × 10^3^ CFU/mL in RPMI 1640 medium (Sigma) supplemented with 0.2% (*w/v*) glucose. One hundred microliter aliquots of these cell suspensions were dispensed into 96-well microtiter plates. Compounds were serially diluted using RPMI 1640 medium and added to the wells at a final concentration ranging from 0.4 to 26 μM, and the plate was incubated for 24 h at 37 °C. Voriconazole (ranged from 8 to 60 μg/mL for ATCC 10231 and *Ca*3 strains; ranged from 0.03 μg/mL to 8 μg/mL for other strains) and Amphotericin B (ranged from 0.12 to 2 μg/mL) were chosen as positive controls.

The MIC was defined as the lowest concentration of the peptide that resulted in 100% growth inhibition after 24 h of incubation. The MFC was determined by transferring 50 μL aliquots of each sample, previously treated with concentrations equal to or higher than the MIC, onto SDA plates and incubating the plates at 37 °C for 24 h. The lowest peptide concentration that yielded no fungal growth on agar plates was defined as the MFC. All the tests were conducted at least three times using independent cell suspensions.

### Checkerboard assay

The interaction between compounds and voriconazole against voriconazole-resistant *C. albicans* strains ATCC 10231 and *Ca*3 clinical strain was evaluated by the checkerboard method in 96-well microtiter plates. Briefly, compounds **Pent-1B** or **Dec-1B** and voriconazole were serially diluted along the y and x axes, respectively. The final concentration of voriconazole ranged from 0.03 to 0.5 µg/mL while the final concentration of **Pent-1B** or **Dec-1B** ranged from 0.8 to 13 μM for ATCC 10231 and from 0.8 to 26 μM for the clinical strain. The checkerboard plates were inoculated with yeast as previously reported and incubated at 37 °C for 24 h, following which yeast growth was assessed visually and the turbidity measured by microplate reader at 595 nm. The Fractional Inhibitory Concentration (FIC) index for each combination was calculated as follows: FIC index = FIC of peptide + FIC of voriconazole, where FIC of peptide (or voriconazole) was defined as the ratio of MIC of peptide (or voriconazole) in combination and MIC of peptide (or voriconazole) alone. The FIC index values were interpreted as follows: ≤0.5, synergistic; >0.5 to ≤1.0, additive; >1.0 to ≤2.0, indifferent; and >2.0, antagonistic effects^9^.

### Peptide cytotoxicity on HaCat cells

To evaluate the effect of **Dec-1B** on HaCaT cell viability, cells were cultured in Petri culture dishes (100 × 20 mm) in Dulbecco’s modified Eagle’s medium (DMEM) supplemented with 10% FBS, 2 mM L-glutamine, 100 U mL^−1^ penicillin, 100 μg mL^−1^ streptomycin, 25 mM HEPES, (Corning) and 130 μg mL^−1^ Na pyruvate in a humidified 5% carbon dioxide atmosphere at 37 °C. Cell viability was examined as previously described^29^. Briefly, using a colorimetric assay based on the MTT labelling reagent, HaCaT cells (1 × 10^4^ cells/well) were seeded in 96-well plates and, after overnight incubation, were treated with Dec-1B at concentrations of 3.25, 6.50, 13.0, 26.0 µM^35^. As internal control, cells containing only dimethylsulphoxide (DMSO) (Sigma-Aldrich D8418-100 ml) as the solvent at the same concentration used in each test wells were selected. After 24 h, 10 µL of MTT solution (5 mg mL^−1^ in phosphate-buffered saline, PBS; pH 7.4) was added to each well and the plates were incubated for 3 h at 37 °C. Then, the medium was removed, and the obtained formazan crystals were dissolved in 150 μL of DMSO for 15 min. The spectrophotometric absorbance was measured using a microtiter enzyme-linked immunosorbent assay reader (Multiskan™ GO Microplate Spectrophotometer; Thermo Scientific™) at 540 nm. The percentage of viability of cells was determined by the following formula: (OD of treated cells/OD of control) × 100. Our *in vitro* cytotoxic examination revealed a secure profile for **Dec-1B** at all tested concentrations on HaCaT cells.

### Critical aggregation concentration

The critical aggregation concentration (CAC) of **Dec-1B** was calculated using Nile Red (NR) as the fluorescent probe. This probe has poor solubility in water, while in presence of hydrophobic environments such as aggregated produces a hyper-hypsochromic effect consisting of a blue-shift and an intensity increase^36^. The peptide stock concentration of 400 µM was prepared dissolving **Dec-1B** in 1,1,1,3,3,3-Hexafluoro-2-propanol (HFIP), and then different peptide aliquots (0.8, 1, 5, 10, 15, 20, 30, 50, 100, 150, 200, 250, 300 µM) were prepared. The organic solvent was removed under nitrogen stream, 0.5 ml of water was added to each peptide aliquots, and then were sonicated for 15 min and freeze-dried^36^. Then, each peptide solution was hydrated with NR solution of 500 nM for 1 h. The NR spectra were recorded at a fluorescence emission between 570 and 700 nm (slit width, 5 nm), and an excitation wavelength of 550 nm (slit width, 10 nm). All data were analysed by plotting the maximum emission fluorescence corresponding wavelength (y) as a function of the peptide concentration (×) using the sigmoidal Boltzmann equation as described previously^29^.

### Thioflavin T assay

Peptide aggregation in presence of LUVs mimicking fungal membrane was monitored by Thioflavin T (ThT) assay^37^. Once lipid film [PE/PC/PI/Ergosterol (5:4:1:2, *w/w/w/w*)] at Cf = 100 μM was prepared as described above, it was hydrated with 100 mM NaCl, 10 mM Tris-HCl, 25 µM ThT buffer for 1 h and then was treated through the extrusion method to obtain LUVs. The LUVs were titrated with different peptide concentrations (5, 10, 20, 30 μM) and the ThT emission spectra were recorded before and after the peptide treatment at a fluorescence excitation at 450 nm (slit width, 10 nm) and a fluorescence emission at 482 nm (slit width, 5 nm).

### Tryptophan quenching by Br-PC

Three different preparations of large unilamellar vesicles (LUVs) containing 25% of phospholipids 6,7 Br-PC, 9,10 Br-PC, 11,12 Br-PC, were prepared to perform tryptophan quenching experiment being the phospholipid Br-PC known as tryptophan quencher^38^. Lipid films composed of PE/PC/Br-PC/PI/Ergosterol (5:3.7:0.3:1:2, *w/w/w/w/w*) were prepared at a final concentration of 125 μM. After the lyophilisation, the film was hydrated with water and LUVs were obtained as described above. The, LUVs were incubated with 5 μM of peptides **Pent-1B** and **Dec-1B** for 2 min and the tryptophan spectra emission was recorded setting the fluorescence excitation at 295 nm.

### Tryptophan quenching by acrylamide

The tryptophan residue of peptides **Pent-1B** and **Dec-1B** was quenched using a 3.25 M solution of acrylamide in the absence and in presence of LUVs composed of PE/PC/PI/Ergosterol (5:4:1:2, *w/w/w/w*) at a final concentration of 250 μM^39^. Once LUVs were prepared as described above, they were incubated with the peptide at 5 μM for 2 min, and then the tryptophan residue was quenched by acrylamide at different concentrations of 0.02, 0.04, 0.06, 0.08, 0.1, 0.12, 0.14, 0.16, 0.18, and 0.2 M. The same quenching experiment was performed for both peptides in water at a concentration of 5 μM. Fluorescence was measured with *λ*_ex_ 295 nm and *λ*_em_ 340 nm. The data were analysed with the Stern-Volmer equation, F_0_/F = 1 + K_sv_ [Q], where F_0_ and F represent the fluorescence intensities in the absence and the presence of the quencher (Q), respectively. The K_sv_ is the Stern-Volmer quenching constant, which is correlated to the accessibility of the tryptophan residue to acrylamide. As acrylamide does not significantly partition into the membrane bilayer, K_sv_ is a reliable reflection of the bimolecular rate constant for collisional quenching of the aromatic residues present in the aqueous phase. K_sv_ is thus determined by the amount of non-lipid-associated free peptide as well as the peptide fraction located on the surface of the bilayer.

### NMR spectroscopy

Appropriate amounts of peptides were dissolved in 0.600 ml H_2_O/D_2_O (90/10 *v/v*) for a peptide concentration of 0.4–0.8 mM to carry out the NMR analysis in pure water. Then, increasing volumes (μL) of a stock DPC-d_38_ solution (1.0 M in H_2_O/D_2_O 90/10 *v/v*, pH 6) were added until no modification was observed on the one-dimensional spectra^40^. The NMR analysis in micellar environments was performed at DPC/peptide molar ratio R of 67, corresponding to DPC concentrations of 20–40 mM and peptide concentrations of 0.3–0.6 mM. Assuming a micelle aggregation number of ∼30, the micelle/peptide molar ratio R is ∼ 2.

NMR spectra were recorded on a Bruker 700 MHz Spectrometer, located at the Department of Pharmacy – University “Federico II” of Naples, and equipped with a z-gradient 5 mm triple-resonance cryoprobe. One-dimensional proton (1 D) and two-dimensional homonuclear (2 D) NMR spectra were recorded at a temperature of 298 K. The spectra were calibrated relative to TSP (0.00 ppm) as an internal standard. 2 D TOCSY (with different mixing times ranging from 25 to 70 ms), and NOESY (mixing times ranging from 100 to 300 ms) spectra were recorded in the phase-sensitive mode using the method from States, using 4096 data points in t2 and 512 equidistant t1 values. The water resonance was suppressed by use of gradients. NMR spectra were analysed by using CARA program (http://cara.nmr.ch/doku.php/home). Proton resonances were sequentially assigned by following the Wuthrich standard method^41^ and are reported in Tables S2–S5 of the Supplementary Material.

**Table 2.** MIC and MFC values of **Pent-1B** and **Dec-1B** against test *C. albicans* strains.

C. albicans strains	Pent-1B		Dec-1B		Voriconazole	Amphotericin B
	MICµM	MFC µM	MIC µM	MFC µM	MIC µM (µg/mL)	MIC µM (µg/mL)
*Ca*1	13	26	>26	nt	1.4 (0.5)	0.54 (0.5)
*Ca*2	6.5	13	>26	nt	1.4 (0.5)	0.13 (0.12)
*Ca*3	26	26	26	26	172 (60)	0.54 (0.5)
*Ca*4	>26	nt	26	26	23 (8)	0.54 (0.5)
*Ca*5	26	26	>26	nt	0.029 (0.01)	2.2 (2)
*Ca*6	>26	nt	26	26	0.086 (0.03)	0.54 (0.5)
*ATCC 10231*	13	13	6.5	6.5	86 (30)	0.13 (0.12)
*ATCC 90028*	13	13	6.5	6.5	0.17 (0.06)	0.13 (0.12)

Conventional antimycotic voriconazole and amphotericin B were used as positive control of antimicrobial activity. nt: not tested.

### Spin-label experiments

Few microliters of a stock solution of 16-doxylstearic acid (16-DSA) in DMSO-d_6_ was added to the DPC peptide samples to obtain a ratio spin-label/micelle 1:1.

### Structure determinations

The NOE-based distance restraints were obtained from NOESY spectra recorded with a mixing time of 200 ms. The NOE cross-peaks were integrated with CARA and were converted into upper distance bounds using CALIBA program^42^ incorporated into CYANA^43^. Geminal protons were chosen as distance reference with 2.0 Å. Three dimensional structures were obtained by using inter-proton distances evaluated from NOEs as upper limits. Structure calculations started from 100 randomised conformers and used the standard CYANA simulated annealing schedule with 20,000 torsion angle dynamics steps per conformer. As the peptide sequences contain modified residues such as D-residues at positions 9 and 10 (d-Leu^9^ and d-Lys^10^) and the N-terminal phenylalanine (Phe) bearing a long aliphatic chain in position para of phenyl ring i.e. pentanoic acid for **Pent-1B** and decanoic acid for **Dec-1B**, all these modifications have been inserted in the special.lib of CYANA suite before calculations. All the conformers showed a fairly agreement with experimental constraints showing no violations.

The best CYANA structures out of 100, in terms of agreement with experimental data, i.e. with lowest target function (TF) values (Table S6), were chosen to represent the peptide molecular structures in solution and visualised by PyMOL software (DeLano (2002) http://www.pymol.org).

### Leakage assay

Large unilamellar vesicles (LUVs) mimicking fungal membrane, PE/PC/PI/Ergosterol (5:4:1:2, *w/w/w/w*), with ANTs and DPX encapsulated, were prepared as previously described.^21^ Firstly, dry lipid film was prepared at a final concentration of 100 µM, and fluorophores ANTs and DPX dissolved in water were added to the film. Then, the lipid preparation was lyophilised overnight. The lipid film loaded with ANTs and DPX was hydrated with PBS 1× buffer (pH= 7.2) for 1 h under stirring and then was treated using the extrusion method to obtain LUVs. In particular, the lipid film with ANTs and DPX was freeze-thawed 6 times and extruded through 0.1-μm pore size polycarbonate filters 10 times. Then, nonencapsulated ANTs and DPX were removed from LUVs through gel filtration using a Sephadex G-50 column (1.5 cm × 10 cm). In the experiment, we measured the dequenching of ANTs by DPX after the treatment of LUVs with different peptide concentrations of 5, 10, 15, 20, 30, and 50 μM for 15 min. The ANTs fluorescence was recorded at a fluorescence excitation at 385 nm (slit width, 5 nm) and a fluorescence emission at 512 nm (slit width, 5 nm). The complete LUVs leakage and release of ANTs were measured after the treatment of LUVs with a solution of 10% Triton-X for 15 min. Liposomes leakage at different peptide concentrations was calculated as follows: %leakage= (F_i_-F_0_)/(F_t_-F_0_), where F_0_ is the ANTs fluorescence of the undamaged LUVs, Fi is the ANTs fluorescence after the peptide addition, and F_t_ is the ANTs fluorescence after the Triton-X treatment.

### Plasma membrane permeabilization assay

The effect of **Pent-1B** and **Dec-1B** on plasma membrane of *C. albicans* cells was investigated by propidium iodide uptake using LIVE/DEAD FungaLight Yeast Viability Kit (Molecular Probes), according to the methodology by Thevissen *et al.* with some modifications^43^. A cell suspension of *C. albicans* ATCC 10231 (200 μL; 3.2 × 10^6^ cells/mL of PBS) was incubated with test peptide at MIC value concentration and live/dead dyes (according to manufacturer instructions) for 30 min and 2 h in microcentrifuges tubes with constant agitation. After this time, the cells were washed, centrifugated (5 min at 10,000 × g) and suspended in 20 μL of PBS. Images were captured using an LSM 710 inverted confocal laser-scanning microscope (Zeiss) equipped with a 63× objective lens.

### Cellular localisation of dec-1B

Confocal laser-scanning fluorescence microscopy was used to study the localisation of **Dec-1B** in *C. albicans* ATCC 10231 cells using a double staining^44^. A cell suspension (200 μL; 3.2 × 10^6^ cells/mL of PBS) was incubated with 150 nM MitoTracker Orange (chloromethyl-H2-tetramethyl rosamine, Molecular Probes), a permanent mitochondrion-selective dye, for 15 min at 37 °C. The cells were washed with 200 μL of PBS and treated for 30 min and 2 h with 6.5 µM of 5(6)-carboxyfluorescein-labelled **Dec-1B**. The cells were centrifugated (5 min at 10,000 × g), suspended in 20 μL of PBS and examined using an LSM 710 confocal laser-scanning microscope with a 63X objective lens. The entire experiment was performed protected from light.

## Results

### Peptide design

**Figure 1.**
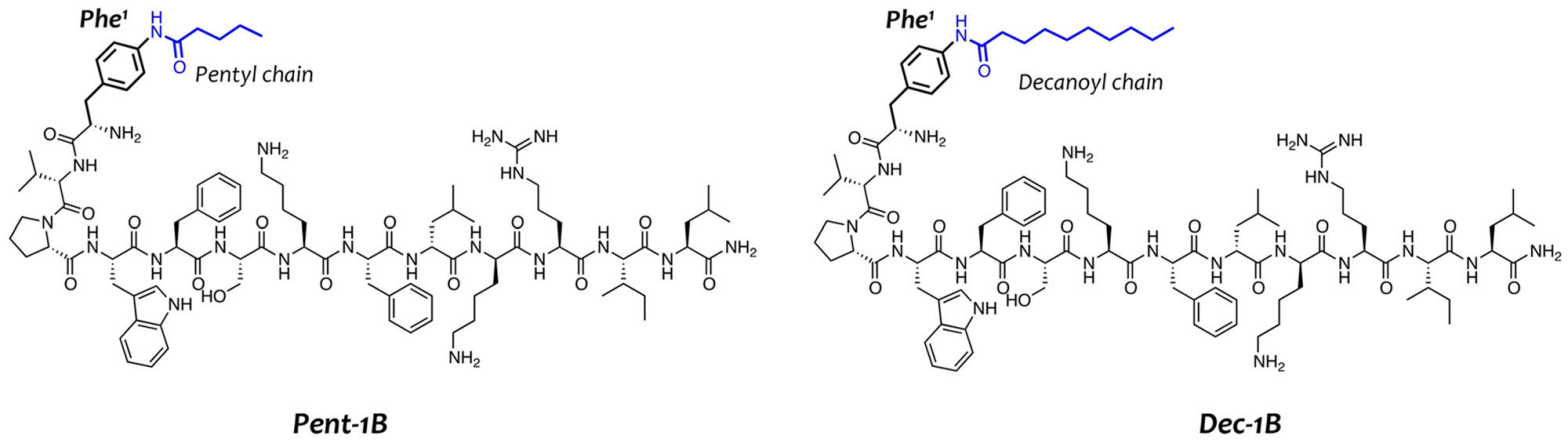
Representation of peptide sequences of **Pent-1B** and **Dec-1B** derived from the lipidation of the peptide **1B**.

Promoting the peptide self-assembly in fungal membrane and reducing the aggregation in aqueous solution through the lipidation strategy could favour an enhancement of the biological activity of AMPs. Different studies have already shown that the addition of fatty acids in N- or C-terminus of AMPs increases their antifungal and antibacterial activities^45^^,^^46^. Starting from our previous lipidation study, we identified the para position of Phe^1^ of peptide **1B** as the best position for the incorporation of lipid tails^29^ because when we conjugated fatty acids of variable length in N-terminus or C-terminus of peptide **1B**, we observed a loss of activity. In this previous study, we discovered a promising lipidated analogue namely **Pent-1B** bearing a lipid tail of 5-carbon atoms (C-5) in the para position of Phe^1^ (Figure 1), which showed a good biological profile in terms of antimicrobial and cytotoxicity activities.

Herein, we explored the incorporation of a longer lipid tail of 10-carbon atoms (C-10) in the para position of Phe^1^ yielding the peptide **Dec-1B** (Figure 1). The aim of this modification was to enhance the vehiculation and anchoring of the peptide within the fungal membrane, attempting to preserve the good biological profile of peptide **Pent-1B**^30^. In our design, we kept unchanged the net positive charge of +4 and the amphipathic properties of the peptide **Pent-1B** and we did not explore the effect of further longer lipid tails to save the right hydrophobic/hydrophilic balance essential for the membrane interaction and to attain a good biological profile.

### Antifungal activity of peptides against *C. albicans* strains

The antimicrobial activity of peptides **Pent-1B** and **Dec-1B** was evaluated against *C. albicans* ATCC 90028 and *C. albicans* ATCC 10231, and six clinical drug-resistant *C. albicans* strains. The antimycotic-susceptibility profiles of clinical *C. albicans* strains are reported in Table 1.

The antifungal activity of peptides, expressed as MIC and MFC values, is shown in Table 2. As reported, both peptides resulted active against both ATCC test strains with MIC values of 13 μM for **Pent-1B** and 6.5 μM for **Dec-1B**. The activity tested on clinical strains showed an inhibitory effect on the growth of 4/6 strains for the peptide **Pent-1B** (MIC values ranged from 26 to 6.5 μM) and of 3/6 strains for the peptide **Dec-1B** at the highest tested concentration of 26 μM. In addition, the activity of peptides was also investigated in terms of minimum fungicidal concentration. The MFC values were equal or two-fold higher than the MIC values for **Pent-1B**, and equal to the corresponding MICs for **Dec-1B**.

### Synergistic effect with voriconazole of acyl-peptides

To further investigate the antifungal activity of **Pent-1B** and **Dec-1B** the synergistic interaction with voriconazole on voriconazole- resistant ATCC 10231 and voriconazole-resistant clinical *Ca*3 strain was determined using the checkerboard technique. After 24 h of incubation we analysed the results to determine the best combination of **Pent-1B** or **Dec-1B** with voriconazole. The total absence of growth of the ATCC 10231 strain was observed for 6.5 μM (1/2 MIC) of **Pent-1B** in combination with 0.06 μg/mL voriconazole (MIC of voriconazole alone was 30 μg/mL on *C. albicans* ATCC 10231). The FIC value of 0.502 was indicative of an additive effect for **Pent-1B**. Regarding **Dec-1B**, the total absence of growth was observed for the values of 3.25 μM (1/2 MIC) for **Dec-1B** in combination with 0.03 μg/mL voriconazole. The FIC value of 0.501 suggested an additive interaction as well. The best interaction on clinical strain *Ca*3 (voriconazole MIC = 60 μg/mL) was obtained at value of 13 μM (1/2 MIC) for **Pent-1B** in combination with 0.06 μg/mL voriconazole (FIC value = 0.501), resulting in an additive effect. Finally, peptide **Dec-1B** showed a synergistic effect at a concentration of 6.5 μM (1/4 MIC) in combination with 0.06 μM of voriconazole (FIC = 0.251) on clinical strain *Ca*3, thus being more effective than **Pent-1B** (Table 3).

**Table 3.** Fractional inhibitory concentration (FIC) index values of peptides on selected *C. albicans* strains.

*C. albicans* strains	Agent	MIC (μM)		FIC index
		Alone	Combination	
*Ca3*	Dec-1B	26	6.5	0.251
	Voriconazole	172	0.172	
	Pent-1B	26	13	0.501
	Voriconazole	172	0.172	
ATCC 10231	Dec-1B	6.5	3.25	0.501
	Voriconazole	86	0.086	
	Pent-1B	13	6.5	0.502
	Voriconazole	86	0.172	

### Peptide effect on HaCaT cell viability

**Figure 2.**
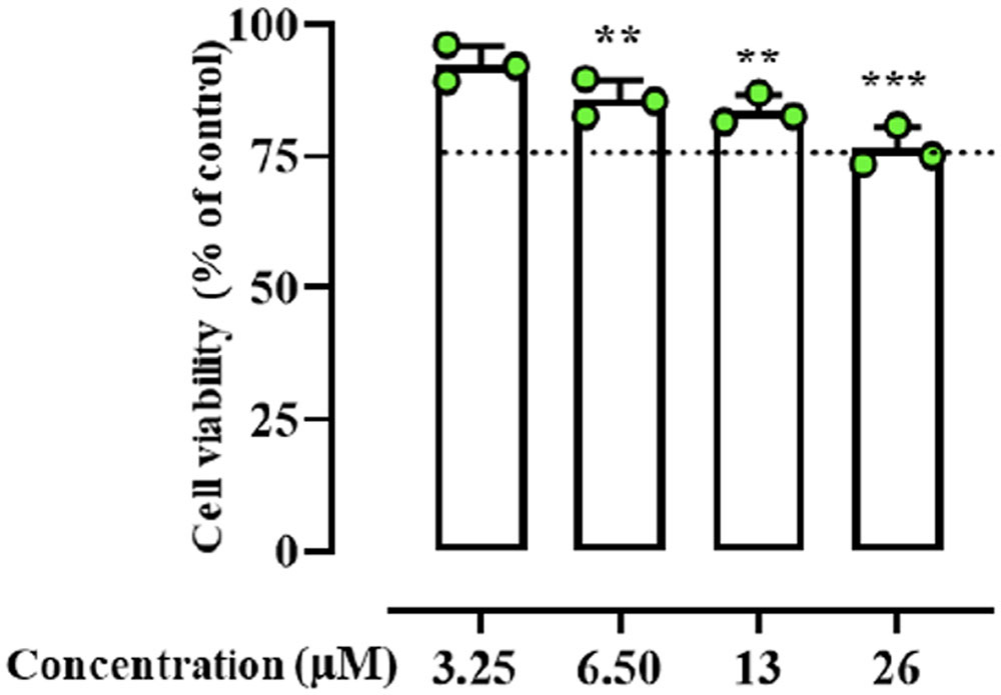
The cell viability of peptide treated HaCaT cells evaluated by MTT assay at 24 h. Dotted lines indicate 75 of cell viability as “cell viability limit”. Values are presented as means ± SD of 3 independent experiments **p ≤ 0.01; ***p ≤ 0.001 vs. Ctrl group. Statistical analysis performed by one way ANOVA followed by Bonferroni’s for multiple comparisons.

Based on the results from previous experiments, the cytotoxic effect of peptide **Dec-1B** was tested on human keratinocytes (HaCat cells) using the 3–(4,5-dimethylthiazol-2-yl)-2,5-diphenyltetrazolium bromide (MTT) assay in a concentration range from 3.25 to 26 µM. As shown in Figure 2, the peptide **Dec-1B** did not produce a significant cytotoxic effect on HaCat cells at its antimicrobial concentration of 6.50 µM. Only at the highest concentration of 26 µM, the incorporation of the lipid tails of C-10 caused a slight increase in the cytotoxicity of **Dec-1B** (75% of cell viability) in comparison with **Pent-1B**, which showed a negligible cytotoxic effect at highest concentration of 25 µM (86.7% of cell viability in Bellavita et al.)^29^.

### Lipid tails promote peptide aggregation in fungal membrane

**Figure 3.**
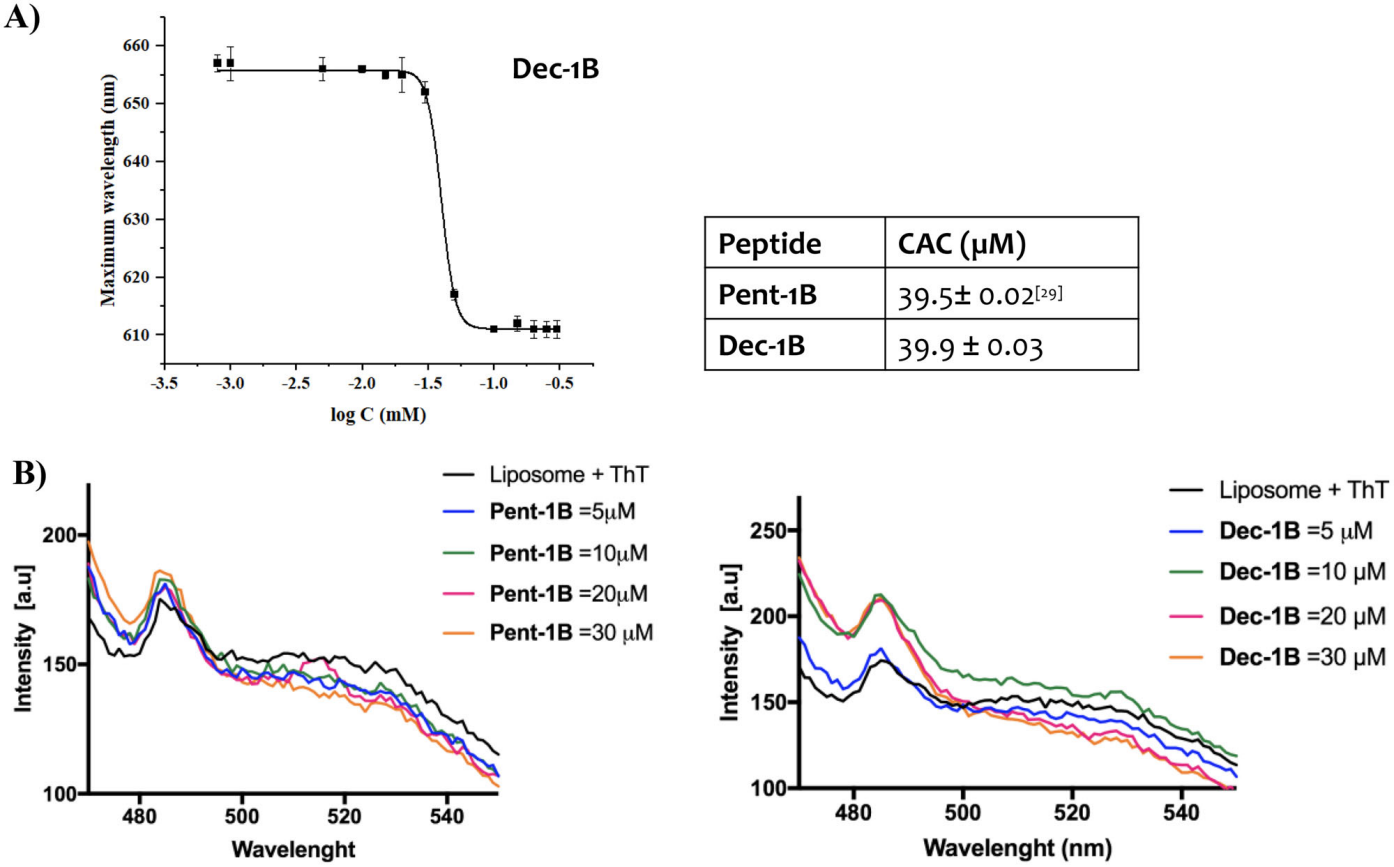
Panel A reports the CAC of the peptide **Dec-1B**, while the CAC of **Pent-1B** was already calculated previously^29^. The CAC was obtained plotting wavelength corresponding to the maximum fluorescence emission of Nile red as a function of the concentration of peptide. Panel B reports the peptide aggregation monitored by ThT fluorescence at different peptide concentrations of 5, 10, 20, and 30 µM.

Lipid tails promote peptide self-assembly in solution as well as in membrane environments. We calculated the critical aggregation concentration (CAC) of peptide **Dec-1B** in aqueous solution using Nile Red (NR) as fluorescent probe (Figure 3, panel A). NR is not highly soluble in water, and it has a large preference to partition into hydrophobic environments producing a blue shift and hyperchromic effect^36^. Despite the presence of the longer lipid tail (C-10), the peptide **Dec-1B** has the same CAC of 39.9 µM of the peptide **Pent-1B**, thus showing similarly a low ability to aggregate in aqueous solution at the concentration used for the antimicrobial assays.

We also explored the ability of the peptides to oligomerize in membrane mimetic environments. The peptide **Dec-1B,** compared to peptide **Pent-1B**, clearly showed a stronger ability to oligomerize in fungal membranes (Figure 3, panel B). Specifically, we studied peptide aggregation in LUVs composed of PE/PC/PI/Ergosterol (5:4:1:2, *w/w/w/w*) mimicking fungal membrane and Thioflavin T as fluorescent dye. LUVs were treated with different peptide concentrations (5, 10, 20, and 30 µM). A large enhancement of ThT was recorded at 10 µM for the peptide **Dec-1B**, revealing a complete peptide aggregation in LUVs. In contrast, for the peptide **Pent-1B**, the ThT fluorescence at different concentrations indicated a progressive phenomenon of aggregation in LUVs, obtaining an increase of ThT fluorescence at the higher concentration of 30 µM.

### Lipopeptides pent-1B and dec-1B are partially inserted into LUVs

The depth of insertion of the peptide into the target membrane is key for activity and for understanding its mechanism of action. We monitored the insertion of lipopeptides **Pent-1B** and **Dec-1B** into LUVs mimicking fungal membrane [PE/PC/PI/Ergosterol (5:4:1:2, *w/w/w/w*)] recording the change of the fluorescence emission of tryptophan (Trp) present in position 4 in both peptides.

**Figure 4.**
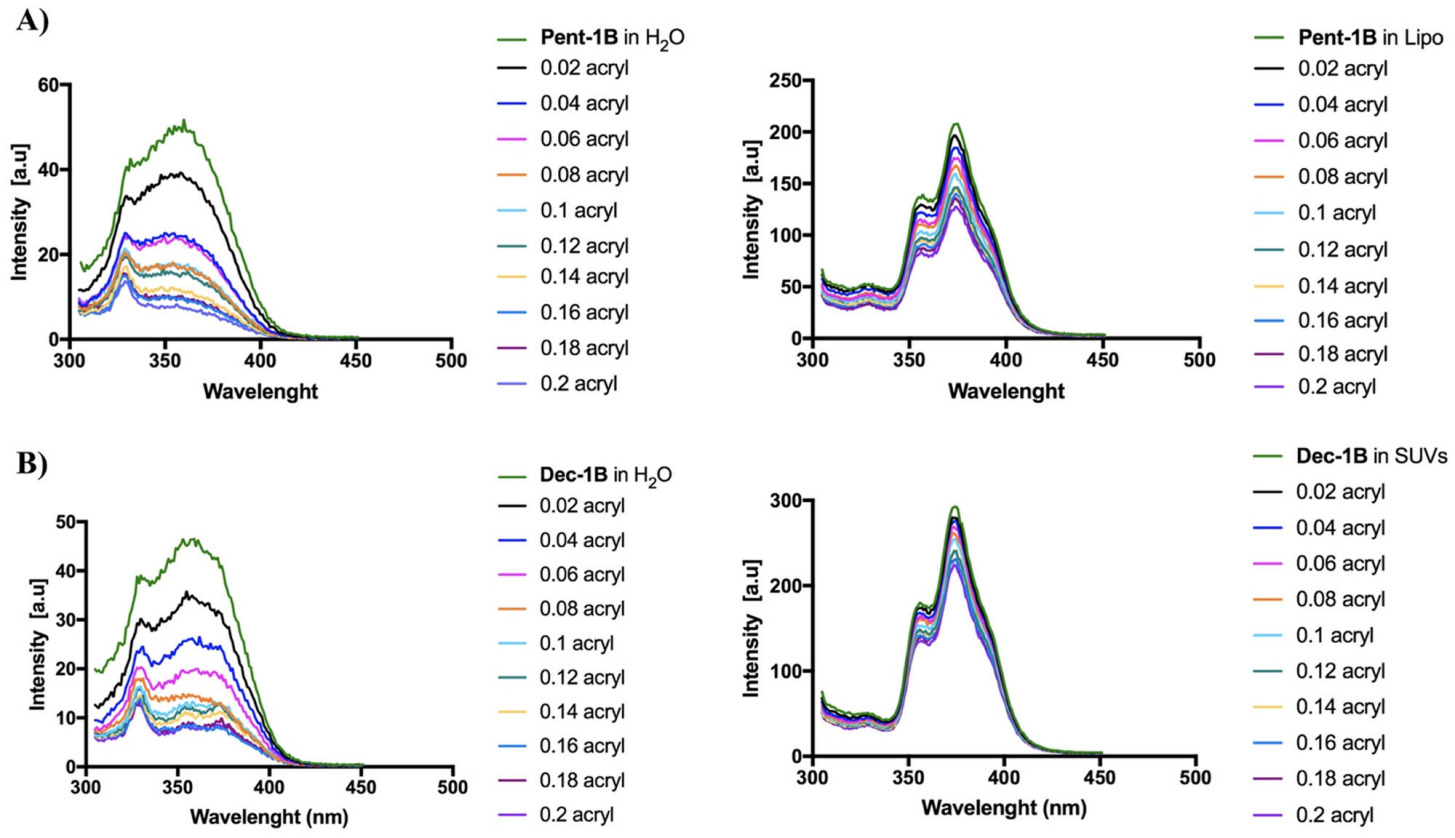
Tryptophan fluorescence spectra for the peptides **Pent-1B** (panel A) and **Dec-1B** (panel B) recorded in water (on the left) and in LUVs composed of PE/PC/PI/Ergosterol (5:4:1:2, *w/w/w/w*) (on the right).

We compared the Trp fluorescence emission of peptides in presence of LUVs and in water; furthermore, the accessibility of the Trp residue was measured by quenching it with acrylamide at different concentrations, which is a water-soluble quencher and is unable to insert into the hydrophobic lipid bilayer. As shown in Figure 4, when the peptides **Pent-1B** and **Dec-1B** are inserted into hydrophobic environments such as LUVs, the quantum yield of Trp residue changes determining an increase of the Trp fluorescence emission with respect to that recorded in water.

The emission spectra in Figure 4 indicated that both peptides (concentration of 10 µM) were buried in LUVs because the Trp residue resulted to be only partially accessible to the quenching by acrylamide at different concentrations. Being both peptides aggregated at the concentration of 10 µM in LUVs as shown in Figure 3, it is likely that equilibrium phenomena take place between monomeric and oligomeric states which make Trp only partially accessible to quenching by acrylamide. In contrast, when the peptides are in aqueous solution, the Trp is highly accessible because both peptides are in monomeric state at 10 µM as indicated by CAC values (Figure 3).

**Figure 5.**
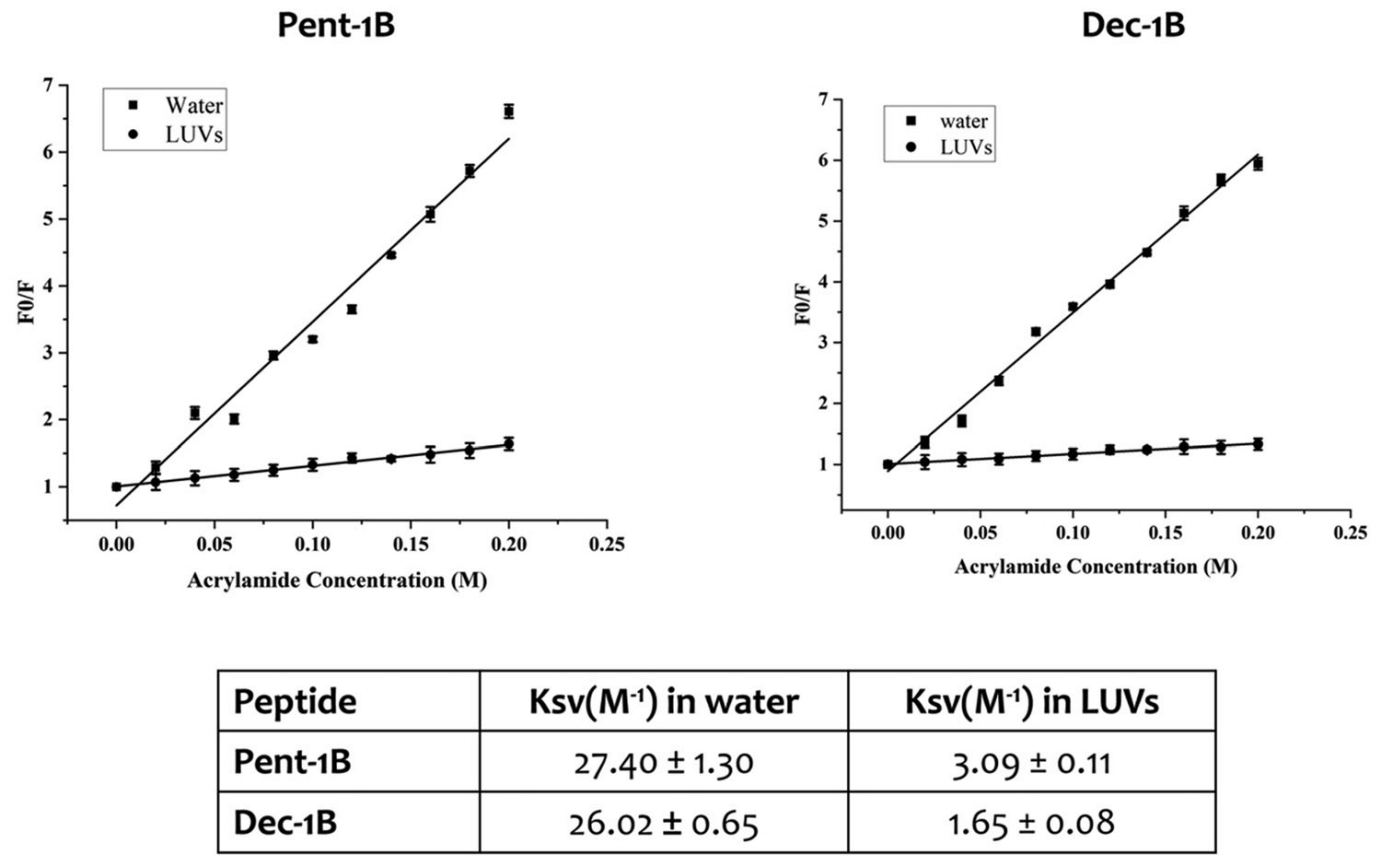
Quenching of tryptophan by acrylamide of peptides **Pent-1B** and **Dec-1B** and Stern-Volmer (Ksv) quenching constants of peptides both in water and LUVs mimicking fungal membrane.

Stern-Volmer plots for the quenching of tryptophan by acrylamide are shown in Figure 5. In the presence of LUVs, a great decrease in fluorescence intensity was evident, thus revealing that the tryptophan is less accessible to the quencher. In fact, the values for K_sv_ were lower (Figure 5) in LUVs, confirming that the tryptophan is more buried in the bilayer.

**Figure 6.**
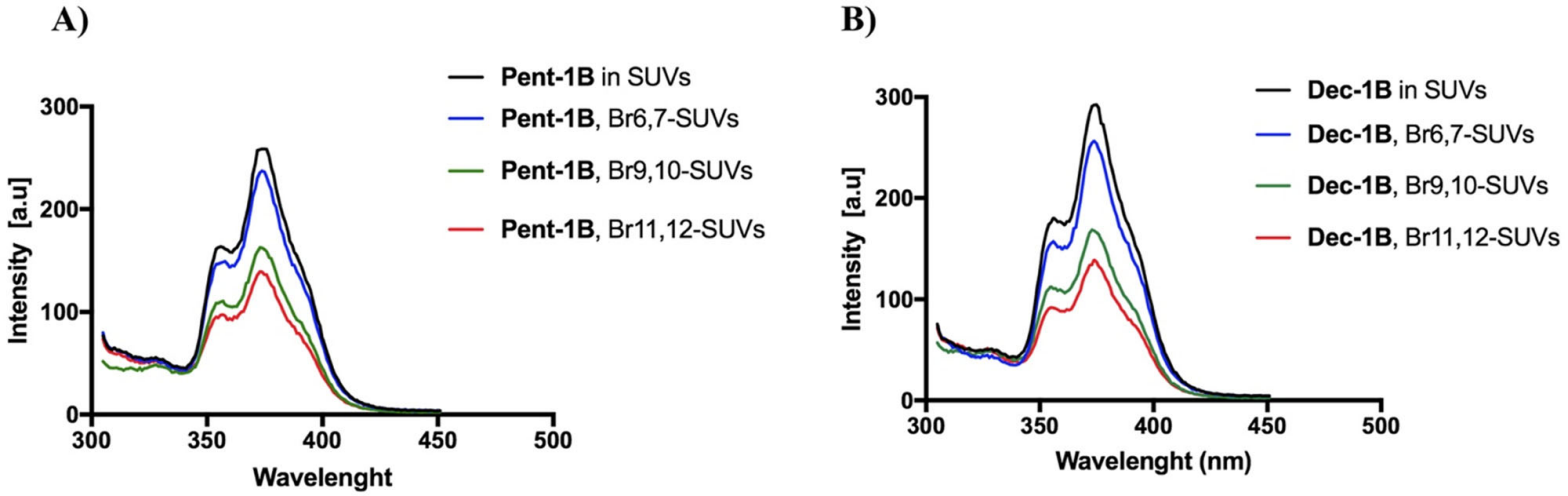
Tryptophan fluorescence spectra for the peptides **Pent-1B** (panel A) and **Dec-1B** (panel B) recorded in the presence of the probes 11,12-Br-PC, 9,10-Br-PC, and 6,7-Br-PC.

In addition, the localisation of both peptides into LUVs mimicking the fungal membrane was investigated by measuring the quenching of the fluorescence of the Trp residue by the bromo probes bound to the lipid chains of the membrane (Figure 6). We used LUVs composed of 3 different phospholipids bearing bromo quencher in different positions along the hydrocarbon chain. In particular, we used phospholipids 11,12-Br-PC, 9,10-Br-PC, and 6,7-Br-PC in our experiment. While in 6,7-Br-PC LUVs, the bromo quencher is located at the LUVs interface, in 11,12-Br-PC and 9,10-Br-PC LUVs, the bromo quencher is deeply buried into the lipid core.^38^ As observed in Figure 5, for both peptides, the largest quenching of Trp fluorescence was recorded in presence of 9,10-Br-PC and 11,12- Br-PC LUVs, while it was less in presence of 6,7- Br-PC LUVs.

These results indicated that probably both peptides were inserted into the lipid bilayer and the Trp residue points towards the micellar interior.

### NMR conformational analysis shows the localisation of lipopeptides into micelle mimicking membrane environment

We further investigated the interaction and structural properties of peptides **Pent-1B** and **Dec-1B** in fungal membranes by NMR spectroscopy using dodecyl-phosphorylcholine (DPC) micelles mimicking membrane environment. NMR studies of **Pent-1B** and **Dec-1B** peptides were performed in pure water, where they are perfectly soluble but poorly structured, and in DPC micelles, where they partially adopt helix structures.

In aqueous medium, NMR data, such as the deviations of αCH proton chemical shift (Figure S1) from random coil values^47^ and NOEs collected for **Pent-1B** and **Dec-1B** in H_2_O/D_2_O (90/10, *v/v)* (Figure S2A and 2C, and Table S6, respectively), indicate random coil conformations of both peptides. This structural diagnosis is confirmed by the molecular models computed by CYANA program^48^ using NOE derived distances as upper limit (upl) of interproton distances sketched in Figure S3. The best ten CYANA structures in terms of agreement with experimental data, *i.e.* with lowest target function (TF) values, were chosen as representative of the conformational space accessible to the peptides and reveal a conformational flexibility of **Pent-1B** and **Dec-1B** in pure water, even though some hints of ordered structural elements, bends or turns, emerge in the middle region of peptides. 1 D ^1^H spectra of the peptides in water solution as well as the CYANA molecular models obtained for **Pent-1B** and **Dec-1B** are shown in Figures S3, S4A and S4B.

The conformational behaviour of **Pent-1B** and **Dec-1B** was also tested in DPC/water mixture (20–40 mM DPC, pH 6, DPC/peptide molar ratio R of 67, DPC micelle/peptide ratio *ca*. 2). 1 D ^1^H spectra of **Pent-1B** and **Dec-1B** in DPC are shown in Figure S3A and S5B. In such environment, both peptides adopt a quite ordered structure. The negative αCH deviations from random coil values < −0.1 ppm (Figure S6) point to a helical conformation in the middle-C-terminal regions of the peptide^46^. This structural diagnosis is consistent with the NOE pattern (Figure S2B, D).

**Figure 7.**
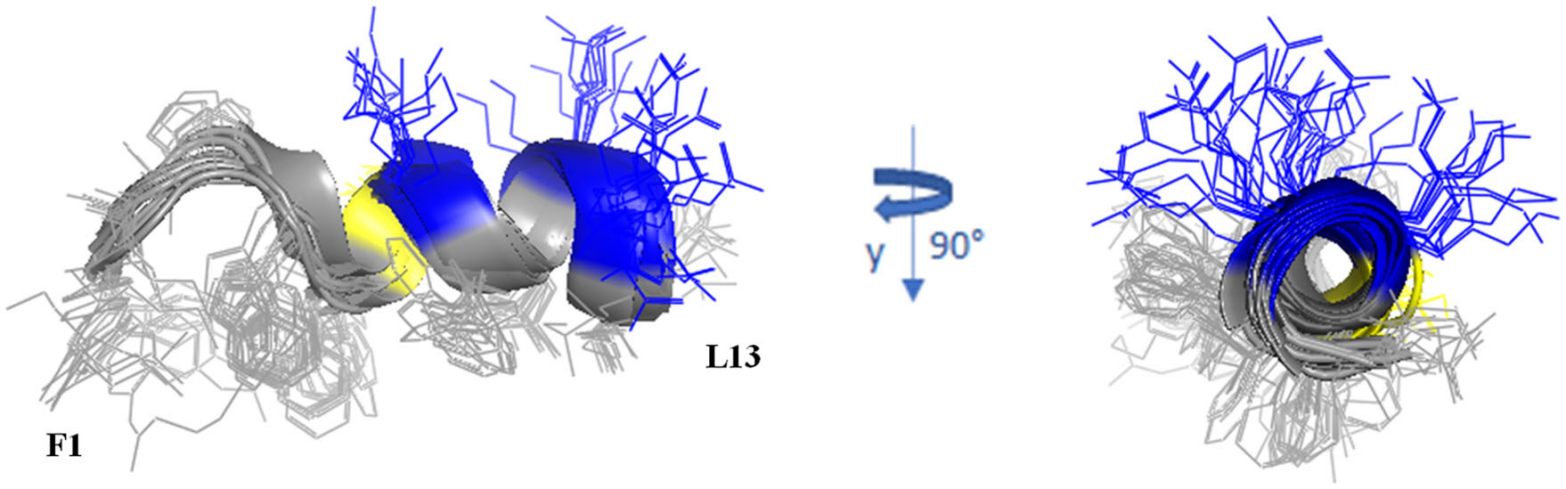
Cartoon representation of the best 15 NMR/CYANA structures of **Pent-1B** in DPC 20 mM. Side - chains are shown as sticks coloured by amino acid type: gray, hydrophobic; blue, basic; yellow, polar.

Indeed, the presence of NOE effects of the type NH_i_–NH_i+1_, together with long range α_i_-N_i+2_, α_i_-N_i+3_ and α_i_-β_i+3_ contacts, indicates the occurrence of a helical structure in the central region of the peptides. Structural calculations of **Pent-1B** and **Dec-1B** were carried out by CYANA^43^ using as upper limit (upl) of inter-proton distances 191 NOE derived distances (127 intra-residues, 33 sequential, 31 long-range) for **Pent-1B** and 246 NOE derived distances (156 intra-residues, 45 sequential, 45 long-range) for **Dec-1B**. The statistic of the structural analysis is reported in Table S6. The best CYANA structures were chosen as representative of the conformational space accessible to the peptide (Figure 7).

**Figure 8.**
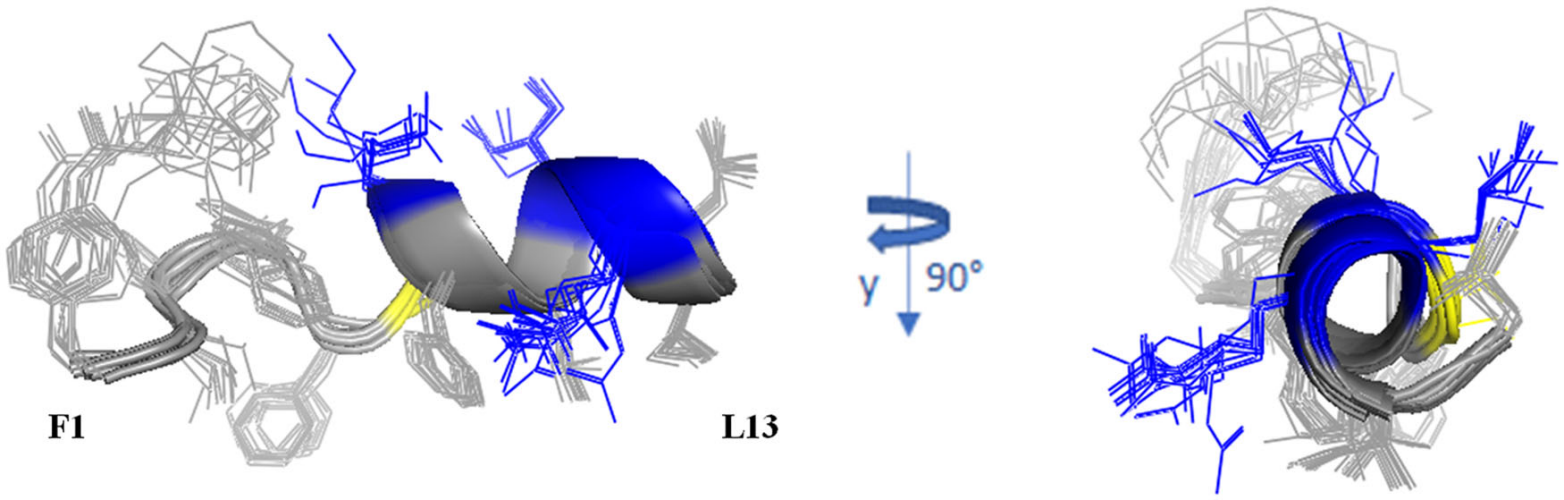
Cartoon representation of the best 15 NMR/CYANA structures of **Dec-1B** in DPC 40 mM. Side - chains are shown as sticks coloured by amino acid type: gray, hydrophobic; blue, basic; yellow, polar.

The backbone superimposition of the best 15 structures shows a well-defined helix arrangement in F^5^–I^12^ segment (RMSD value on the backbone atoms of 0.52 Å) that places positively charged and hydrophobic side chains on opposite sides. It is worth noticing that, in this arrangement, all the charged sidechains (K^7^ K^10^ R^11^) are iso-oriented as well as the aromatic side chains of N-terminal residues F^1^ W^4^ and F.^5^ Similarly, for **Dec-1B** the NMR/CYANA analysis finds a helical arrangement in the segment 5–12 (RMSD value on the backbone atoms of 0.25 Å), with iso-orientation of the hydrophobic and charged side chains (Figure 8).

### Spin label studies

The spin label 16-doxyl stearic acid (16-DSA) was added to the peptide/DPC samples to examine the placement of peptides in DPC. 16-DSA is useful for identification of the membrane-embedded residues, as it solubilises into DPC micelles^49^. This spin label contains one unpaired electron that induces paramagnetic relaxation resulting in line broadening of resonance signals in proximity to the spin label. A comparison of the cross-peak intensities in the TOCSY spectra of peptide/micelle systems with and without the spin label indicates the residues mostly interacting with micelles.

With a 16-DSA/DPC micelle ratio of 1:1 the cross-peak intensities of the N-terminal residues are strongly reduced, whilst the cross-peak intensities of the C-terminal residues show the same periodic medium or strong reduction for **Pent-1B** as well as for **Dec-1B**. The least affected residues are serine 6 and lysine 7 at the beginning of the α-helix structure.

**Figure 9.**
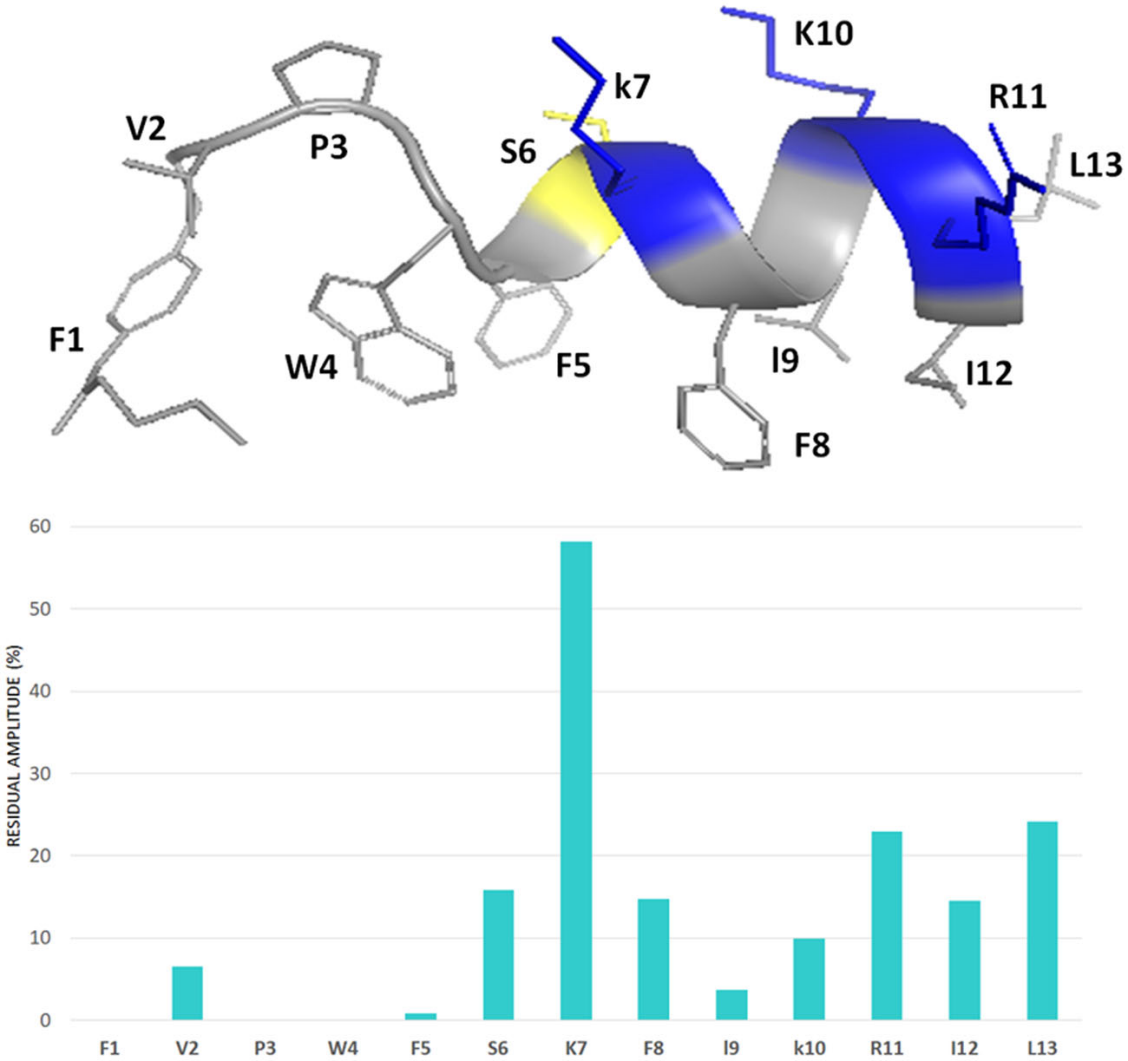
The percentage of signal intensity remaining after the addition of the 16-DSA to **Pent-1B** in DPC for HN—Hα TOCSY cross peaks.

These results suggest that helical domain lies parallel to the micelle surface, with the hydrophobic face towards the interior of the micelle, and the positively charged residues interacting with the negatively charged phosphate headgroups, anchoring the peptide just below the micelle surface (Figure 9).

### Lipopeptides pent-1B and dec-1B did not induce membrane leakage

Since different studies showed that temporins generally act causing the breakdown of bacterial membranes^22^^,^^29^, we investigated the mode of action of lipopeptides **Pent-1B** and evaluated the release of encapsulated ANTs probe in LUVs mimicking fungal membrane [PE/PC/PI/Ergosterol (5:4:1:2, *w/w/w/w*)]. In these conditions, we did not observe any ANTs release and liposome leakage at different peptide concentrations of 5, 10, 15, 20, 30, and 50 µM (data not shown). Thus, we hypothesised that the mode of action of these lipopeptides may involve an initial electrostatic interaction followed by aggregation and insertion in the lipid bilayer causing perturbation through a carpeting effect as already shown for some temporin analogues^21^ and other molecular mechanisms are surely underlying their antifungal activity.

### Lipopeptides cause yeast membrane permeabilization

**Figure 10.**
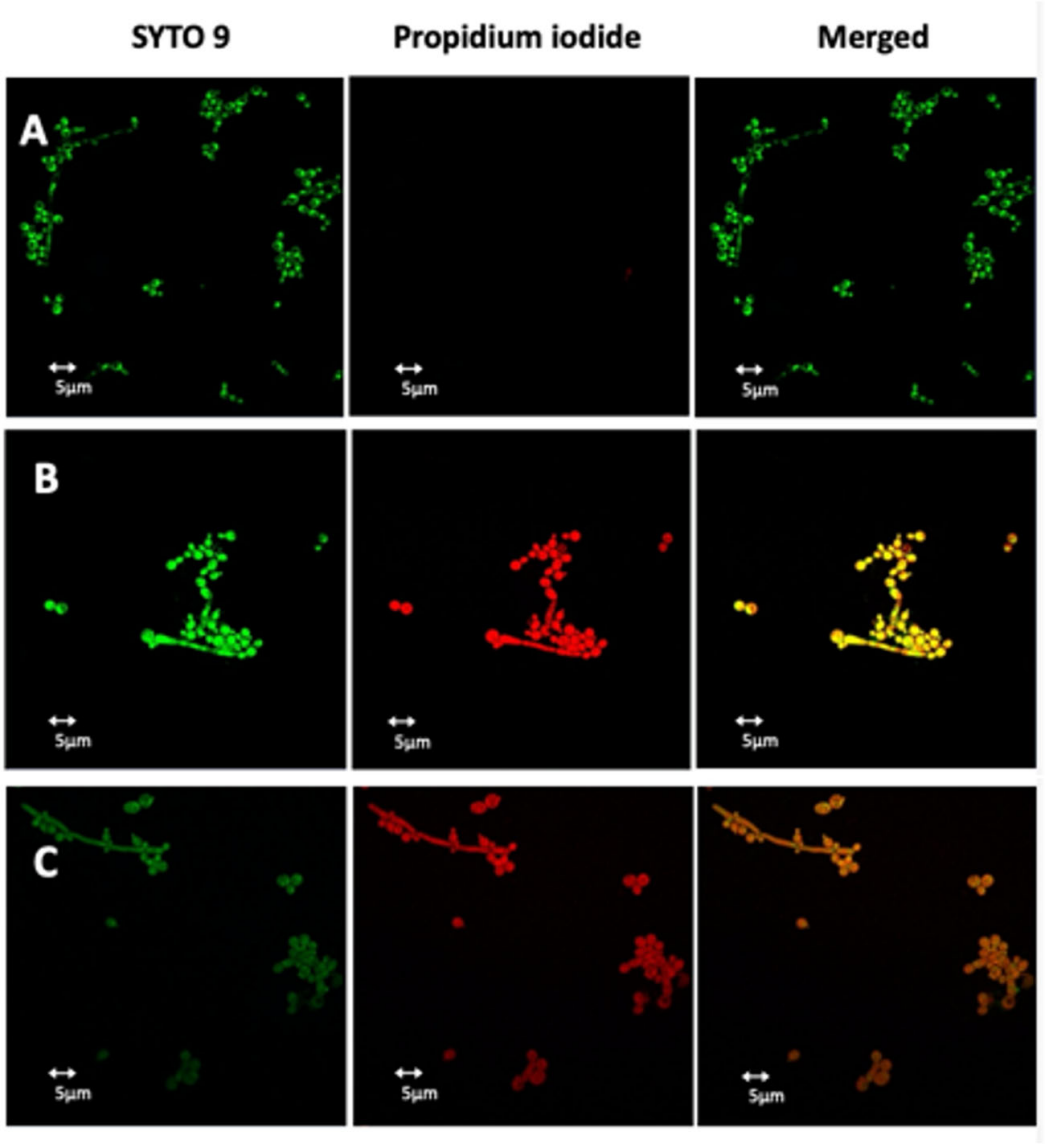
Detection of cell membrane permeabilisation in living *C. albicans* cells from control culture (A) and cultures treated with **Pent-1B** (B) or **Dec-1B** (C) by CLSM. The cells were stained with SYTO9 and Propidium iodide (PI). Fluorescence images of the same samples at 528 nm for SYTO9 (green fluorescence; left panels), 645 nm for PI (red fluorescence; middle panels) and merged images (right panels) are shown. SYTO 9 penetrates both viable and nonviable cells, while PI penetrates only cells with compromised membranes. *Candida* cells showing yellow-red fluorescence are considered as dead cells.

To evaluate the membrane permeabilization of peptides **Pent-1B** and **Dec-1B,** *C. albicans* cells were treated with the LIVE/DEAD FungaLight Yeast Viability kit consisting of labelling dyes such as SYTO 9 and propidium iodide (PI). SYTO 9 penetrates both viable and non-viable cells, while PI only penetrates cells with damaged membranes by turning off the fluorescence emitted by SYTO 9. Damaged or dead cells emit a yellow-red fluorescence, while viable cells emit only green fluorescence. The images were captured by an LSM710 inverted confocal laser scanning microscope (CLSM). All yeast cells showed yellow-red fluorescence after 30 min of treatment with **Pent-1B** or **Dec-1B** peptides (Figure 10, panels B and C), while untreated cells showed green fluorescence (Figure 10, panel A). The obtained results further support the data obtained by the other experiments and pointing on the ability of both peptides to act on the plasma membrane of these cells, causing a membrane perturbation and thus allowing the entry of the PI dye.

### Dec-1B does not enter in yeast mitochondria

**Figure 11.**
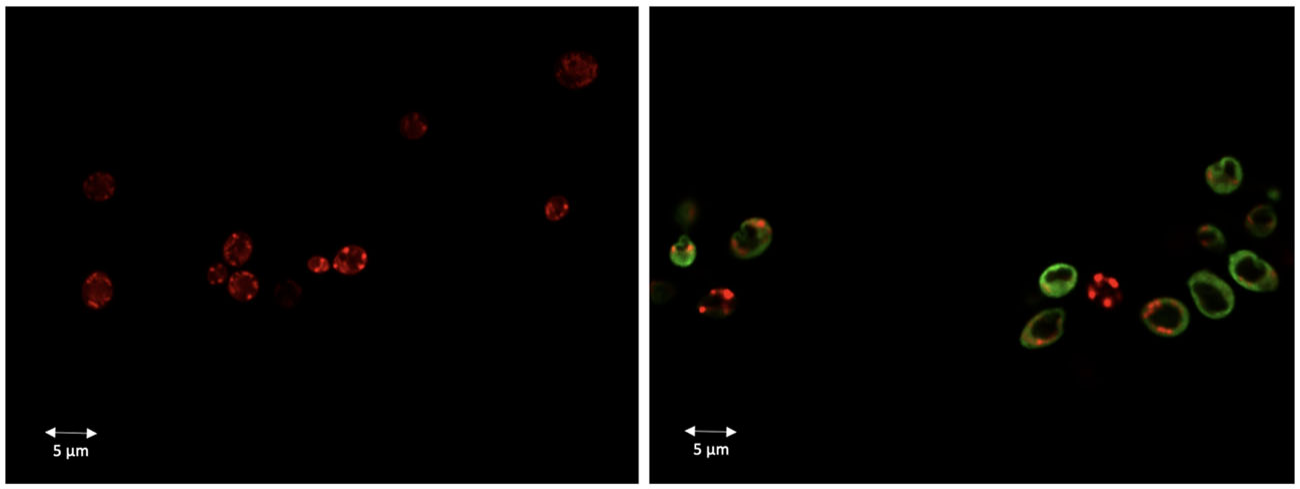
**Dec-1B** localisation in *C. albicans* cells detected by CLSM. **Dec-1B** conjugated with 5(6)-carboxyfluorescein is coloured in green, while the mitochondria are visualised in red by MitoTracker. The images show *C. albicans* cells before treatment (left panel) and after treatment for 30 min with **Dec-1B** (right panel) with the green signal localised on the surface.

**Dec-1B** labelled with 5(6)-carboxyfluorescein was used to investigate the cellular localisation of the peptide in *C. albicans* cells using CLSM. A suspension of the ATCC 10231 strain was pre-incubated with MitoTracker Orange and then treated with the labelled peptide at the MIC value concentration. Confocal images showed that the green signal corresponding to the localisation of the fluorescent peptide was uniformly localised on the surface of the treated cells after 30 min. Since the peptide signal has not been shown to overlap the red signal (MitoTracker) of the mitochondria (Figure 11), no localisation in mitochondria seems to occur. The green signal remained localised on yeast surface even after 2 h of treatment. The images therefore confirm that the peptide acts on the fungal wall/cell membranes.

## Discussions

The spread of *Candida* infections and the resistance issues to available antifungal drugs are rapidly increasing. In this scenario, AMPs represent a challenge to open novel avenues and hopes for overcoming the antifungal resistance^14^^,^^16^. Previously, using several synthetic strategies on some Temporin L derivatives, including lipidation strategy^29^ we developed different analogues which exhibited strong ability to kill both Gram-positive and Gram-negative bacteria, and yeasts as *Candida* spp^21^. Our previous lipidation study, consisting in the conjugation of fatty acids at the *N-* and *C-*terminus of the most promising Temporin L analogue, peptide **1B**, allowed to identify the para position of Phe^1^ as the best position for performing this functionalization^29^. In this lipidation screening, we discovered the acylated peptide **Pent-1B** bearing the acyl chain of C-5 on Phe^1^ which displayed a strong activity against carbapenem resistant *Klebsiella pneumoniae* (MIC = 6.25 µM) and a low cytotoxic profile^29^^,^^30^. Through a deep study on its potential mode of action, we observed that the presence of the chain of the C-5 in **Pent-1B** determined a monomeric state in aqueous solution (CAC= 39.5 µM) and a significant ability to oligomerize at a low concentration (∼10 µM) in LUVs mimicking Gram-positive and Gram-negative membranes^29^. Following the oligomerization in bacterial membrane, **Pent-1B** induced the pore formation of the target membrane detected by a complete leakage of LUVs mimicking bacterial membrane.

Here, considering these encouraging results, we further investigated the activity of **Pent-1B** against reference and clinical strains of *Candida* and we designed and developed its analogue **Dec-1B** (Figure 1), bearing a longer lipid tail of C-10 in para position of Phe^1^ to improve peptide anchoring within the fungal membrane.

In general, the interaction of AMPs with the target membranes is regulated by several features, including the length of the sequence, the number and density of positive charges, the secondary structure in aqueous environment and in membranes, and the hydrophobicity^50^. The initial interaction involves the binding to the membrane by a purely non-specific electrostatic interaction between the positively charged residues of the peptide and the negatively charged membrane of bacteria or fungi. Whereas, the peptide hydrophobic domains interact with the hydrophobic core of the lipid bilayer, guiding the peptide deeper into the membrane^50^^,^^51^.

In our design, we kept the net positive charge of +4 of peptide **Pent-1B** due to Lys and Arg residues and added a longer lipid tail of C-10 to boost the peptide interaction and the internalisation into a complex membrane like that of fungi. The results of antifungal activity on *C. albicans* ATCC strains (ATCC 10231 and ATCC 90028) showed a significant antifungal activity for **Pent-1B** and **Dec-1B** with a MIC of 13 and 6.5 µM, respectively (Table 2). Both peptides were able to reduce the growth of *C. albicans* clinical strains, with MIC values ranging from 26 μM to 6.5 μM for peptide **Pent-1B** on *Ca*1, *Ca*2, *Ca*3 and *Ca*5 strains (Table 2), while the peptide **Dec-1B** worked at the highest tested concentration of 26 μM on 3 strains (*Ca*3*, Ca*4*, Ca*6). Although in most cases MIC values for clinical strains were higher than those obtained for ATCC strains, these results seem interesting as the peptides were effective against clinical strains with different resistance profiles, some of which very difficult to treat. In particular, *Ca*3 and *Ca*6 strains were resistant to all the azoles, which are widely used for the topical treatment of superficial candidiasis and the systemic therapy of invasive mycoses, while the *Ca*1 and *Ca*6 strains were resistant to both azoles and echinocandins, which frequently represent the therapeutic option for azole-resistant *Candida* strains in hospital settings^52^.

Currently, combination treatment of antifungal drugs with unconventional antifungal agents have been proposed to fight drug-resistant *C. albicans* infections^53^. In our study, when combined with voriconazole, **Dec-1B** exhibited a synergistic effect on *Ca*3 strain, while the association had an additive effect on resistant ATCC strain (Table 3). On the other hand, the combination of **Pent-1B** with voriconazole was always additive. Noteworthy is the ability of both peptides to significantly reduce the MIC of voriconazole for both resistant strains ATCC 10231 and *Ca*3, thereby restoring their susceptibility to the antifungal drug. In this case, the combination approach seems particularly advantageous as it allows to recover the effectiveness of drugs such as azoles which have limited toxicity, are cheap and available for oral administration.

Based on these synergistic results, we hypothesised that both peptides may act with different mode of actions compared to conventional antifungal drugs, facilitating the entry of azoles due to their ability to enhance the cell membrane permeability. Clearly, we still cannot exclude that the peptides may also act on some specific molecular pathways and further studies will be necessary to demonstrate this latter mechanism.

We previously showed that **Pent-1B** acts through a membrane-active mechanism on bacteria^29^ thus, here, we explored the mode of action of peptides **Pent-1B** and **Dec-1B** through fluorescence assays using liposomes as model systems for mimicking fungal membrane and NMR studies to analyse their three-dimensional structure.

Both peptides were in a monomeric state in aqueous solution at their active antifungal concentrations (around 10 µM) detected by the value of CAC (around 39 µM). Whereas, both peptides showed the ability to oligomerize in liposomes mimicking the *Candida* membrane detected by the ThT experiment (Figure 3). In particular, the peptide **Dec-1B** bearing the longer lipidic tails oligomerized at lower concentration of 10 µM than the peptide **Pent-1B** that is completely aggregated at 30 µM.

We also analysed through the Br-Trp quenching experiment the depth of internalisation of the peptides in LUVs and the results supported that the tryptophan of both peptides was strongly inserted in the bilayer (Figure 6). The tryptophan is located at position 4 of the sequence from the N-terminus, which confirms the hypothesis that the flexible domain at the N-terminus is key for the insertion into the bilayer.

Since the other important feature that influences the mode of action of AMPs is three-dimensional structure, we studied the potential mode of action of both peptides by NMR spectroscopy. We observed that both peptides are disordered in water but partially organised in helix when interacting with membrane mimetic systems. The choice of the mimetic system is a critical point in this type of study. We opted for a micellar system instead of bicelles or other heavier mimics because micelles are generally light enough to allow the use of un-labelled (neither ^15^N and/or ^13^C labelling) cheaper peptides for the NMR interactions studies. The addition of DPC to each **Pent-1B** and **Dec-1B in** aqueous solution modifies the NMR spectra. The lines of all signals become larger and some amide protons belonging to the *N-*terminal segment vanish at all. These effects, by alone, demonstrate that the peptides bind the micelles. Indeed, both peptides change their roto-translation correlation times from those typical of the free forms to those, longer, of the micelle, with consequent higher relaxation rates and larger or vanished signals. Temporin analogue ability to interact with membrane mimics with consequent adoption of helix structure was previously demonstrated^21^^,^^22^^,^^29^. Here, we showed that the pentanoic or decanoic acid covalently linked to the *para* position of Phe^1^ for **Pent-1B** and **Dec-1B**, respectively, favours the *N-*terminal anchorage of the peptides to the micelle.

For both peptides, the hydrophobic trait, TAIL-F^1^-V^2^-P^3^-W,^4^ deeply binds the micelle, while the trait F^5^-L^13^ adopts an α-helix structure. The helix arrangement is driven by the contact between the 5–13 trait, amphiphilic in nature, and micelle. The recourse to radical species like 16-doxyl stearic acid (16-DSA), makes it possible to catch the NMR imagine of **Pent-1B** and **Dec-1B** docked to the micelle surface. DSA, which solubilises into the micelle, accelerates the nuclear relaxation rates of the nuclei around it. That means that more the peptide side chains are sunk into the micelle, more their signal intensities are diminished. For both **Pent-1B** and **Dec-1B**, the nuclei with the most decreased intensities are those of the N-terminal lipophilic segments and those of the first 3–4 residues. The effect of the paramagnetic species on the intensity of the segment 5–13 suggests that the helix lies on the micelle surface, with the aromatic side chains of both **Pent-1B** (Figure 9) and **Dec-1B** iso-oriented towards the micelle interior, while the positively charged sidechains protrude on the opposite side. Thus, the picture emerging by NMR measurements points to peptides that are deeply anchored through the N-terminal segment, while the C-terminal trait lies on the micelle surface fastened by the hydrophobic side chains.

The Br-Trp quenching experiment in membranes mimicking the fungal membrane clearly showed a deeper insertion of the peptides compared to the NMR results obtained into micelles mimicking mammalian membranes, further supporting that the antifungal activity associated by the lack of toxicity to mammalian cells is associated to a different depth of penetration.

The results of the NMR studies in combination with biophysical data clearly showed that both peptides have the typical behaviour of AMPs, characterised by a random conformation in aqueous solution and the adoption of a helical conformation in membrane bilayers. Furthermore, the presence of the lipidic chain favours the peptide anchorage to the membrane and the formation of oligomers inside the membrane. In addition, the deep insertion and the absence of leakage are key to hypothesise a carpet mechanism for the perturbation of the membrane. This assumed mechanism of action of the peptides was further investigated by CLSM. *Candida* membrane permeabilisation assay showed that 30 min of treatment with **Pent-1B** or **Dec-1B** (separately) allowed the uptake of PI suggesting that wall/cell membrane damage and permeabilisation is a possible mode of action of these lipopeptides (Figure 10). In agreement with these results, the fluorescent labelling of **Dec-1B-K(Fam)** showed that it remained uniformly localised at the surface of *Candida* cells and was not localised into mitochondria (Figure 11).

These data support the hypothesis that **Pent-1B** and **Dec-1B** act by interacting and altering the fungal cell membrane as already reported for many antimicrobial peptides, although other mechanisms of action cannot be excluded.

## Conclusions

In conclusion, this study elucidated the antifungal activity of our acylated Temporin L analogues **Pent-1B** and **Dec-1B** and explored their potential membrane active mechanism. The acylated peptides resulted to be efficacious on both reference and clinical isolates of *C. albicans* and they showed an additive or synergistic interaction with voriconazole. In addition, both peptides did not show cytotoxicity on human keratinocytes at their antimicrobial concentration. Through a deep study on their potential mechanism of action by using fluorescence-based assays, NMR studies and confocal microscopy, we hypothesised a membrane-active mechanism for peptides **Pent-1B** and **Dec-1B**, that consists in an internalisation and oligomerization into fungal membrane, causing a cell membrane damage by a carpeting effect.

These findings may provide insights into the possible therapeutic applications of Temporin analogues as antifungal agents or as enhancers of traditional antifungal drugs that are losing their efficacy due to the development of resistance.

## Supplementary Material

Supplemental Material

## References

[1] Domán M, Bányai K. COVID-19-associated fungal infections: an urgent need for alternative therapeutic approach.? Front Microbiol. 2022;13:919501. 35756020 10.3389/fmicb.2022.919501 PMC9218862

[2] Ré ACS, Martins JF, Cunha-Filho M, Gelfuso GM, Aires CP, Gratieri T. New perspectives on the topical management of recurrent candidiasis. Drug Deliv Transl Res. 2021;11(4):1568–1585. 33469892 10.1007/s13346-021-00901-0

[3] Mukaremera L, Lee KK, Mora-Montes HM, Gow NAR. *Candida albicans* yeast, pseudohyphal, and hyphal morphogenesis differentially affects immune recognition. Front Immunol. 2017;8(1):629. 10.3389/fimmu.2017.00629 PMC5461353 28638380

[4] de Cássia Orlandi Sardi J, de Souza Pitangui N, Gullo FG, Fusco-Almeida AM, Mendes-Giannini MJS. A mini review of *Candida* species in hospital infection: epidemiology, virulence factor and drugs resistance and prophylaxis. Trop. Med. Surg. 2013;1:5.

[5] Nucci M, Queiroz-Telles F, Tobón AM, Restrepo A, Colombo AL. Epidemiology of opportunistic fungal infections in Latin America. Clin Infect Dis. 2010;51(5):561–570. 20658942 10.1086/655683

[6] Talapko J, Juzbašić M, Matijević T, Pustijanac E, Bekić S, Kotris I, Škrlec I. *Candida albicans*-the virulence factors and clinical manifestations of infection. JoF. 2021;7(2):79. 33499276 10.3390/jof7020079 PMC7912069

[7] Naglik JR, Challacombe SJ, Hube B. *Candida albicans* secreted aspartyl proteinases in virulence and pathogenesis. Microbiol Mol Biol Rev. 2003;67(3):400–428, table of contents. 12966142 10.1128/MMBR.67.3.400-428.2003 PMC193873

[8] Bhattacharya S, Sae-Tia S, Fries BC. *Candidiasis* and mechanisms of antifungal resistance. Antibiotics (Basel. 2020;9(6):312. 10.3390/antibiotics9060312 PMC7345657 32526921

[9] Danby CS, Boikov D, Rautemaa-Richardson R, Sobel JD. Effect of pH on *in vitro* susceptibility of *Candida glabrata* and *Candida albicans* to 11 antifungal agents and implications for clinical use. Antimicrob Agents Chemother. 2012;56(3):1403–1406. 22232293 10.1128/AAC.05025-11 PMC3294902

[10] Zhang MR, Zhao F, Wang S, Lv S, Mou Y, Yao CL, Zhou Y, Li FQ. Molecular mechanism of azoles resistant *Candida albicans* in a patient with chronic mucocutaneous candidiasis. BMC Infect. Dis. 2020;20:126. 32046674 10.1186/s12879-020-4856-8 PMC7014776

[11] Tverdek FP, Kofteridis D, Kontoyiannis DP. Antifungal agents and liver toxicity: a complex interaction. Expert Rev anti Infect Ther. 2016;14(8):765–776. 27275514 10.1080/14787210.2016.1199272

[12] Buda De Cesare G, Cristy SA, Garsin DA, Lorenz MC. Antimicrobial peptides: a new frontier in antifungal therapy. mBio. 2020;11(6):20. 10.1128/mBio.02123-20 PMC7642678 33144376

[13] Wang J, Dou X, Song J, Lyu Y, Zhu X, Xu L, Li W, Shan A. Antimicrobial peptides: promising alternatives in the post feeding antibiotic era. Med Res Rev. 2019;39(3):831–859. 30353555 10.1002/med.21542

[14] Falanga A, Maione A, La Pietra A, de Alteriis E, Vitale S, Bellavita R, Carotenuto R, Turrà D, Galdiero S, Galdiero E, et al. Competitiveness during dual-species biofilm formation of *Fusarium oxysporum* and *Candida albicans* and a novel treatment strategy. Pharmaceutics. 2022;14(6):1167. 35745740 10.3390/pharmaceutics14061167 PMC9227787

[15] Jenssen H, Hamill P, Hancock RE. Peptide antimicrobial agents. Clin Microbiol Rev. 2006;19(3):491–511, 16847082 10.1128/CMR.00056-05 PMC1539102

[16] Maione A, Bellavita R, de Alteriis E. d, Galdiero S, Albarano L, La Pietra AL, Guida M, Parrilli E, D’Angelo C, Galdiero E, et al. A. WMR peptide as antifungal and antibiofilm against *albicans* and non-*albicans Candida* species: shreds of evidence on the mechanism of action. IJMS. 2022;23(4):2151. 35216270 10.3390/ijms23042151 PMC8879636

[17] Silva ARP, Guimarães MS, Rabelo J, Belén LH, Perecin CJ, Farías JG, Santos JHPM, Rangel-Yagui CO. CO. Recent advances in the design of antimicrobial peptide conjugates. J Mater Chem B. 2022;10(19):3587–3600. 35262120 10.1039/d1tb02757c

[18] Romero SM, Cardillo AB, Martínez Ceron MC, Camperi SA, Giudicessi SL. Temporins: an approach of potential pharmaceutic candidates. Surg Infect. 2020;21(4):309–322. 10.1089/sur.2019.266 31804896

[19] Zannella C, Chianese A, Palomba L, Marcocci ME, Bellavita R, Merlino F, Grieco P, Folliero V, De Filippis A, Mangoni ML, et al. Broad-Spectrum antiviral activity of the amphibian antimicrobial peptide Temporin L and its analogs. IJMS. 2022;23(4):2060. 35216177 10.3390/ijms23042060 PMC8878748

[20] Mangoni ML, Grazia AD, Cappiello F, Casciaro B, Luca V. Naturally occurring peptides from *Rana temporaria*: antimicrobial properties and more. Curr Top Med Chem. 2016;16(1):54–64. 26139114 10.2174/1568026615666150703121403

[21] Bellavita R, Maione A, Merlino F, Siciliano A, Dardano P, De Stefano L, Galdiero S, Galdiero E, Grieco P, Falanga A. Antifungal and antibiofilm activity of cyclic Temporin L peptide analogues against *albicans* and non-*albicans Candida* species. Pharmaceutics. 2022;14(2):454. 35214187 10.3390/pharmaceutics14020454 PMC8877061

[22] Bellavita R, Casciaro B, Di Maro S, Brancaccio D, Carotenuto A, Falanga A, Cappiello F, Buommino E, Galdiero S, Novellino E, et al. First-in-Class cyclic Temporin L analogue: design, synthesis, and antimicrobial assessment. J Med Chem. 2021;64(15):11675–11694. 34296619 10.1021/acs.jmedchem.1c01033 PMC8389922

[23] Merlino F, Carotenuto A, Casciaro B, Martora F, Loffredo MR, Di Grazia A, Yousif AM, Brancaccio D, Palomba L, Novellino E, et al. Glycine-replaced derivatives of [Pro^3^,Dleu^9^]TL, a temporin L analogue: evaluation of antimicrobial, cytotoxic and hemolytic activities. Eur J Med Chem. 2017;139:750–761. 28863356 10.1016/j.ejmech.2017.08.040

[24] Bellavita R, Vollaro A, Catania MR, Merlino F, De Martino L, Nocera FP, Della Greca M, Lembo F, Grieco P, Buommino E. Novel antimicrobial peptide from Temporin L in the treatment of *Staphylococcus Pseudointermedius* and *Malassezia Pachydermatis* in polymicrobial inter-kingdom infection. Antibiotics (Basel. 2020;9(9):530. 10.3390/antibiotics9090530 PMC7560154 32842593

[25] Bellavita R, Raucci F, Merlino F, Piccolo M, Ferraro MG, Irace C, Santamaria R, Iqbal AJ, Novellino E, Grieco P, et al. Temporin L-derived peptide as a regulator of the acute inflammatory response in zymosan-induced peritonitis. Biomed Pharmacother. 2020;123:109788–109788. 31865142 10.1016/j.biopha.2019.109788

[26] Hamill RJ. Amphotericin B formulations: a comparative review of efficacy and toxicity. Drugs. 2013;73(9):919–934. 23729001 10.1007/s40265-013-0069-4

[27] de Alteriis E, Maione A, Falanga A, Bellavita R, Galdiero S, Albarano L, Salvatore MM, Galdiero E, Guida M. Activity of free and liposome-encapsulated essential oil from *Lavandula angustifolia* against persister-derived biofilm of *Candida auris*. Antibiotics. 2021;11(1):26. 35052903 10.3390/antibiotics11010026 PMC8772840

[28] Allen TM, Cullis PR. Liposomal drug delivery systems: from concept to clinical applications. Adv Drug Deliv Rev. 2013;65(1):36–48. 23036225 10.1016/j.addr.2012.09.037

[29] Bellavita R, Falanga A, Buommino E, Merlino F, Casciaro B, Cappiello F, Mangoni ML, Novellino E, Catania MR, Paolillo R, et al. Novel Temporin L antimicrobial peptides: promoting self-assembling by lipidic tags to tackle superbugs. J Enzyme Inhib Med Chem. 2020;35:751–1764. 10.1080/14756366.2020.1819258 PMC7534258 32957844

[30] Roscetto E, Bellavita R, Paolillo R, Merlino F, Molfetta N, Grieco P, Buommino E, Catania MR. Antimicrobial activity of a lipidated Temporin L analogue against carbapenemase-producing *Klebsiella pneumoniae* clinical isolates. Antibiotics. 2021;10(11):1312. 34827250 10.3390/antibiotics10111312 PMC8614721

[31] Clinical and Laboratory Standards Institute (CLSI). Performance standards for antifungal susceptibility testing of yeasts. M60 – 2nd Ed. Pennsylvania (US): Clinical and Laboratory Standards Institute; 2020.

[32] The European Committee on Antimicrobial Susceptibility Testing - EUCAST. Clinical breakpoints for fungi (Candida and Aspergillus species). Available from: https://www.eucast.org/astoffungi/clinicalbreakpointsforantifungals. v. 10.0, accessed on 4 April 2022.

[33] Merlino F, Tomassi S, Yousif AM, Messere A, Marinelli L, Grieco P, Novellino E, Cosconati S, Maro D. S. Boosting Fmoc solid-phase peptide synthesis by ultrasonication. Org Lett. 2019;21(16):6378–6382. 31361506 10.1021/acs.orglett.9b02283

[34] Clinical and Laboratory Standards Institute. Reference method for broth dilution antifungal susceptibility testing of yeasts. M27 – 4th Ed. Pennsylvania (US): Clinical and Laboratory Standards Institute; 2017.

[35] Varma SR, Sivaprakasam TO, Mishra A, Prabhu S, M R, P R. Imiquimod-induced psoriasis-like inflammation in differentiated human keratinocytes: its evaluation using curcumin. Eur J Pharmacol. 2017;813:33–41. 28736282 10.1016/j.ejphar.2017.07.040

[36] Del Genio V, Falanga A, Allard-Vannier E, Hervé-Aubert K, Leone M, Bellavita R, Uzbekov R, Chourpa I, Galdiero S. Design and validation of nanofibers made of self-assembled peptides to become multifunctional stimuli-sensitive nano-vectors of anticancer drug doxorubicin. Pharmaceutics. 2022;14(8):1544. 35893800 10.3390/pharmaceutics14081544 PMC9331957

[37] Xue C, Lin TY, Chang D, Guo Z. Thioflavin T as an amyloid dye: fibril quantification, optimal concentration and effect on aggregation. R Soc Open Sci. 2017;4(1):160696. 28280572 10.1098/rsos.160696 PMC5319338

[38] Bolen EJ, Holloway PW. Quenching of tryptophan fluorescence by brominated phospholipid. Biochemistry. 1990;29(41):9638–9643. 2271606 10.1021/bi00493a019

[39] De Kroon AI, Soekarjo MW, De Gier J, De Kruijff B. The role of charge and hydrophobicity in peptide-lipid interaction: a comparative study based on tryptophan fluorescence measurements combined with the use of aqueous and hydrophobic quenchers. Biochemistry. 1990;29(36):8229–8240. 2252886 10.1021/bi00488a006

[40] Yousif AM, Ingangi V, Merlino F, Brancaccio D, Minopoli M, Bellavita R, Novellino E, Carriero MV, Carotenuto A, Grieco P. Urokinase receptor derived peptides as potent inhibitors of the formyl peptide receptor type 1-triggered cell migration. Eur J Med Chem. 2018;143:348–360. 29202399 10.1016/j.ejmech.2017.11.030

[41] Wuthrich K. NMR of proteins and nucleic acids. New York: Wiley; 1986.

[42] Güntert P, Qian YQ, Otting G, Müller M, Gehring W, Wüthrich K. Structure determination of the Antp (C39––S) homeodomain from nuclear magnetic resonance data in solution using a novel strategy for the structure calculation with the programs DIANA, CALIBA, HABAS and GLOMSA. J Mol Biol. 1991;217(3):531–540. 1671604 10.1016/0022-2836(91)90755-u

[43] Thevissen K, Terras FR, Broekaert WF. Permeabilization of fungal membranes by plant defensins inhibits fungal growth. Appl Environ Microbiol. 1999;65(12):5451–5458. 10584003 10.1128/aem.65.12.5451-5458.1999 PMC91743

[44] Ruissen AL, Groenink J, Helmerhorst EJ, Walgreen-Weterings E, Van’t Hof W, Veerman EC, Nieuw Amerongen AV. Nieuw Amerongen AV. effects of histatin 5 and derived peptides on *Candida albicans*. Biochem J. 2001;356(Pt 2):361–368. 11368762 10.1042/0264-6021:3560361 PMC1221846

[45] Bugli F, Massaro F, Buonocore F, Saraceni PR, Borocci S, Ceccacci F, Bombelli C, Di Vito M, Marchitiello R, Mariotti M, et al. Design and characterization of myristoylated and non-myristoylated peptides effective against *Candida* spp. clinical isolates. IJMS. 2022;23(4):2164. 35216297 10.3390/ijms23042164 PMC8875392

[46] Malina A, Shai Y. Conjugation of fatty acids with different lengths modulates the antibacterial and antifungal activity of a cationic biologically inactive peptide. Biochem J. 2005;390(Pt 3):695–702. 15907192 10.1042/BJ20050520 PMC1199663

[47] Wishart DS, Sykes BD, Richards FM. Relationship between nuclear magnetic resonance chemical shift and protein secondary structure. J Mol Biol. 1991;222(2):311–333. 1960729 10.1016/0022-2836(91)90214-q

[48] Guntert P. Automated NMR structure calculation with CYANA. Methods Mol. Biol. 2004;278:353–378. 15318003 10.1385/1-59259-809-9:353

[49] Brown LR, Bosch C, Wuthrich K. Location and orientation relative to the micelle surface for glucagon in mixed micelles with dodecylphosphocholine: EPR and NMR studies. Biochim. Biophys. Acta. 1981;642(2):296–312. 6269613 10.1016/0005-2736(81)90447-8

[50] Hancock RE, Rozek A. Role of membranes in the activities of antimicrobial cationic peptides. FEMS Microbiol Lett. 2002;206(2):143–149. 11814654 10.1111/j.1574-6968.2002.tb11000.x

[51] Shai Y. Mechanism of the binding, insertion and destabilization of phospholipid bilayer membranes by alpha-helical antimicrobial and cell non-selective membrane-lytic peptides. Biochim Biophys Acta. 1999;1462(1–2):55–70. 10590302 10.1016/s0005-2736(99)00200-x

[52] Pristov KE, Ghannoum MA. Resistance of *Candida* to azoles and echinocandins worldwide. Clin Microbiol Infect. 2019;25(7):792–798. 30965100 10.1016/j.cmi.2019.03.028

[53] Liu S, Hou Y, Chen X, Gao Y, Li H, Sun S. Combination of fluconazole with non-antifungal agents: a promising approach to cope with resistant Candida albicans infections and insight into new antifungal agent discovery. Int J Antimicrob Agents. 2014;43(5):395–402. 24503221 10.1016/j.ijantimicag.2013.12.009

