# Supplementary material for: Unveiling the mechanism of action of acylated temporin L analogues against multidrug-resistant Candida albicans
Source: J Enzyme Inhib Med Chem. 2022 Oct 28;38(1):36–50. doi: 10.1080/14756366.2022.2134359 (PMC9621209; doi:10.1080/14756366.2022.2134359)
Supplement: Supplemental Material [file IENZ_A_2134359_SM1700.pdf]

Supporting information

Unveiling the mechanism of action of Acylated Temporin L analogues against multidrug-resistant Candida albicans

Rosa Bellavita,[a] Annarita Falanga[b], Francesco Merlino,[a] Gabriella D'Auria,[a] Nicola Molfetta,[a] Anella Saviano,[a] Francesco Maione,[a] Umberto Galdiero,[c] Maria Rosaria Catania,[c] Stefania Galdiero,[a] Paolo Grieco,[a] Emanuela Roscetto,[c*] Lucia Falcigno,[a*] Elisabetta Buommino[a]

[a]Department of Pharmacy, University of Naples "Federico II", Via Domenico Montesano 49, 80138 Naples, Italy

[b]Department of Agricultural Science, University of Naples "Federico II", via Università 100, 80055, Portici, Italy

[c]Department of Molecular Medicine and Medical Biotechnology, University of Naples Federico II, Via S. Pansini 5, 80131 Naples, Italy.

Table of contents

1. HPLC chromatograms and ESIMS of peptides **Pent-1B, Dec-1B and DEC-1B-K(Fam)** (Table S1) 3

2. Proton chemical shifts (ppm) of **Pent-1B** in H_2O/D_2O 90/10 at 298 K referred to TSP. (Table S2) 3

3. Proton chemical shifts (ppm) of **Pent-1B** in DPC R67 at 298 K referred to TSP. (Table S3) 4

4. Proton chemical shifts (ppm) of **Dec-1B** in H_2O/D_2O 90/10 at 298 K referred to TSP. **(**Table S4) 5

5. Proton chemical shifts (ppm) of **Dec-1B** in DPC R67 at 298 K referred to TSP. **(**Table S5) 6

6. CYANA structural statistic of **Pent-1B** and **Dec-1B** in free and micelle bound forms (Table S6) 7

7. Chemical shift deviations from random coil values of αCH protons of **Pent-1B** and **Dec-1B** in H_2O/D_2O. (Figure S1) 8

8. Most relevant NOE contacts of peptides **Pent-1B** and **Dec-1B** in different media (Figure S2) 8

9. 700 MHz 1D ^1H spectra of **A)Pent-1B** and B) **Dec-1B** in H_2O/D_2O. (Figure S3) 9

10. Cartoon representation of the best 10 NMR/CYANA structures in H_2O/D_2O of peptides **A)Pent-1B** and B) **Dec-1B** (Figure S4) 9

11. 700 MHz 1D ^1H spectra of A)**Pent-1B** and B) **Dec-1B** in DPC (Figure S5) 10

12. Chemical shift deviations from random coil values of αCH protons of **Pent-1B** and **Dec-1B** in DPC, DPC/peptide molar ratio equal to 67.(Figure S6). 11

Table S1. Analytical and ESIMS data of peptides **Pent-1B**, **Dec-1B** and **Dec-1B-K(Fam)**.

Peptide	Ritention time (t_R)	Mass calculated	Mass found
**Pent-1B**	14.4	1779.2	$[M+3H]^+/3$=594.2
**Dec-1B**	15.9	1848.2	[M+3H]+/3=617.6
**Dec-1B-K(Fam)**	15.1	2334.3	[M+3H]+/3=779.9

Table S2. Proton chemical shifts (ppm) of **Pent-1B** in H_2O/D_2O 90/10 at 298 K referred to TSP.

Residue	NH	αH	βH	γH	others
**Phe[1]**	--	4.29	3.19, 3.09		HD 7.18, HE 7.37*
**Val[2]**	8.32	4.34	1.81	0.83	
**Pro[3]**	--	4.28	1.94, 1.69	2.14	δCH_2 3.54, 3.66
**Trp[4]**	7.75	4.57	3.27, 3.21		2H 7.19; 4H 7.56; 5H 7.25; 6H 7.14; 7H 7.51; NH 10.16
**Phe[5]**	7.58	4.49	2.87		HD 7.07, HE 7.30
**Ser[6]**	7.99	4.30	3.81, 3.74		
**Lys[7]**	8.16	4.23	1.67	1.21	δCH_2 1.62; εCH_2 2.90
**Phe[8]**	8.02	4.56	3.11, 2.99		HD 7.25, HE 7.37
**DLeu[9]**	8.01	4.19	1.49	1.16	δCH_3 0.82, 0.76
**DLys[10]**	8.25	4.23	1.45	1.38	δCH_2 1.79; εCH_2 2.97
**Arg[11]**	8.00	4.32	1.81, 1.70	1.54	δCH_2 3.13; εNH 7.14
**Ile[12]**	8.23	4.13	1.84	1.45	γCH_3 1.16, δCH_3 0.88
**Leu[13]**	8.26	4.32	1.60	1.60	δCH_3 0.86

* Side chain modified $NH\text{-}CO\text{-}CH_2CH_2CH_2CH_3$: 9.72, 2.31, 1.61, 1.33, 0.89 ppm

$CONH_2$ ter 7.49, 7.04 ppm.

Table S3. Proton chemical shifts (ppm) of **Pent-1B** in DPC R67 at 298 K referred to TSP.

Residue	NH	αH	βH	γH	others
**Phe[1]**	--	--	3.14, 3.04		HD 7.21, HE 7.57*
**Val[2]**	--	4.31	1.92	0.85	
**Pro[3]**	--	4.52	2.31, 1.88	2.01	δCH_2 3.68, 3.86
**Trp[4]**	--	4.43	3.34, 3.17		2H 7.42; 4H 7.34; 5H 6.90; 6H 7.03; 7H 7.46; NH 10.78
**Phe[5]**	--	4.34	2.96, 2.87		HD 7.22, HE 7.13
**Ser[6]**	8.30	4.16	3.95, 3.90		
**Lys[7]**	8.09	4.19	1.76	1.27	δCH_2 1.58; εCH_2 2.90
**Phe[8]**	8.06	4.45	3.06		HD 7.22, HE 7.14
**DLeu[9]**	8.60	3.82	1.69	1.46	δCH_3 0.85, 0.75
**DLys[10]**	8.41	3.91	1.98, 1.80	1.40	δCH_2 1.68; εCH_2 2.95
**Arg[11]**	8.10	4.12	1.93	1.69	δCH_2 3.25, 3.19
**Ile[12]**	7.70	4.00	1.89	1.36, 1.18	γCH_3 0.87, δCH_3 0.77
**Leu[13]**	8.00	4.26	1.67	1.74	δCH_3 0.91

*Side chain modified NH-CO-$CH_2CH_2CH_2CH_3$: 9.98, 2.24, 1.57, 1.30, 0.89 ppm. $CONH_2$ ter 7.25 ppm.

Table S4. Proton chemical shifts (ppm) of **Dec-1B** in H_2O/D_2O 90/10 at 298 K referred to TSP.

Residue	NH	αH	βH	γH	others
**Phe[1]**	--	4.29	3.23, 3.06		HD 7.18, HE 7.38*
**Val[2]**	8.30	4.33	1.80	0.82	
**Pro[3]**	--	4.29	2.14, 1.94	1.71	δCH_2 3.56, 3.66
**Trp[4]**	7.74	4.56	3.26, 3.20		2H 7.18; 4H 7.50; 5H 7.24; 6H 7.14; 7H 7.57; NH 10.17
**Phe[5]**	7.58	4.50	2.87		HD 7.07, HE 7.29
**Ser[6]**	7.99	4.31	3.82, 3.75		
**Lys[7]**	8.17	4.21	1.66	1.20	δCH_2 1.58; εCH_2 2.89
**Phe[8]**	8.03	4.56	3.11, 2.98		HD 7.24, HE 7.37
**DLeu[9]**	8.00	4.20	1.49	1.18	δCH_3 0.82, 0.76
**DLys[10]**	8.27	4.24	1.66	1.44, 1.37	δCH_2 1.79; εCH_2 2.96
**Arg[11]**	8.01	4.34	1.81, 1.69	1.54	δCH_2 3.12; εNH 7.15
**Ile[12]**	8.24	4.14	1.84	1.46, 1.15	γCH_3 0.88, δCH_3 0.85
**Leu[13]**	8.26	4.32	1.57	1.22	δCH_3 0.89, 0.82

* Side chain modified NH-CO$(CH_2)_8CH_3$: 9.73, 2.29, 1.61, 1.27, 0.83 ppm
$CONH_2$ ter 7.50, 7.05 ppm.

Table S5. Proton chemical shifts (ppm) of **Dec-1B** in DPC R67 at 298 K referred to TSP.

Residue	NH	αH	βH	γH	others
**Phe[1]**	--	4.25	3.32, 3.02		HD 7.18, HE 7.58*
**Val[2]**	8.32	4.30	1.88	0.89, 0.83	
**Pro[3]**	--	4.58	2.30	1.95, 1.83	δCH_2 3.65, 3.83
**Trp[4]**	--	4.45	3.24, 3.05		2H 7.35; 4H 7.34; 5H 6.88; 6H 7.03; 7H 7.46; NH 10.70
**Phe[5]**	7.61	4.10	3.22, 3.15		HD 7.10, HE 7.19
**Ser[6]**	8.38	4.10	3.89, 3.84		
**Lys[7]**	8.13	4.16	1.75	1.24	δCH_2 1.59; εCH_2 2.88; εNH 7.61
**Phe[8]**	8.07	4.45	3.08, 3.04		HD 7.08, HE 7.18
**DLeu[9]**	8.56	3.78	1.66	1.41	δCH_3 0.80, 0.70
**DLys[10]**	8.42	3.87	1.94, 1.76	1.37	δCH_2 1.64; εCH_2 2.92 εNH 7.69
**Arg[11]**	8.06	4.09	1.91	1.67	δCH_2 3.16
**Ile[12]**	7.68	3.96	1.86	1.32, 1.14	γCH_3 0.84, δCH_3 0.75
**Leu[13]**	7.95	4.23	1.67	1.61	δCH_3 0.88

*Side chain modified NH-CO$(CH_2)_8CH_3$: 9.94, 2.01, 1.42, 1.11 1.08, 0.82 ppm.

$CONH_2$ ter 7.22 ppm.

Table S6. CYANA structural statistic of **Pent-1B** and **Dec-1B** in free and micelle bound forms

	**Pent-1B in water**	**Pent-1B in DPC***	**Dec-1B in water**	**Dec-1B in DPC***
distance restraints	169	191	169	246
intraresidue	128	127	138	156
sequential (|i - j| = 1)	33	33	29	45
medium-range (1< |i - j| ≤ 4)	8	31	2	45
**Violation statistics (100 structures)**				
CYANA TF (Å^2)	0.30 ± 0.39	2.20 ± 1.76	0.037 ± 0.0.26	0.30± 0.34
**Residual Distance Constraint Violations (Å)**				
number > 0.2 Å	0	0	0	0
mean global backbone RMSD	2.98 ± 0.46Å	1.43 ± 0.48Å	2.94 ± 0.59Å	0.74 ± 0.75Å
mean global heavy atom	4.74 ± 0.44 Å	2.50 ± 0.66 Å	4.78 ± 0.55Å	1.68 ± 0.94Å
[illegible]	[illegible]	[illegible]	[illegible]	

*R DPC/peptide = 67.

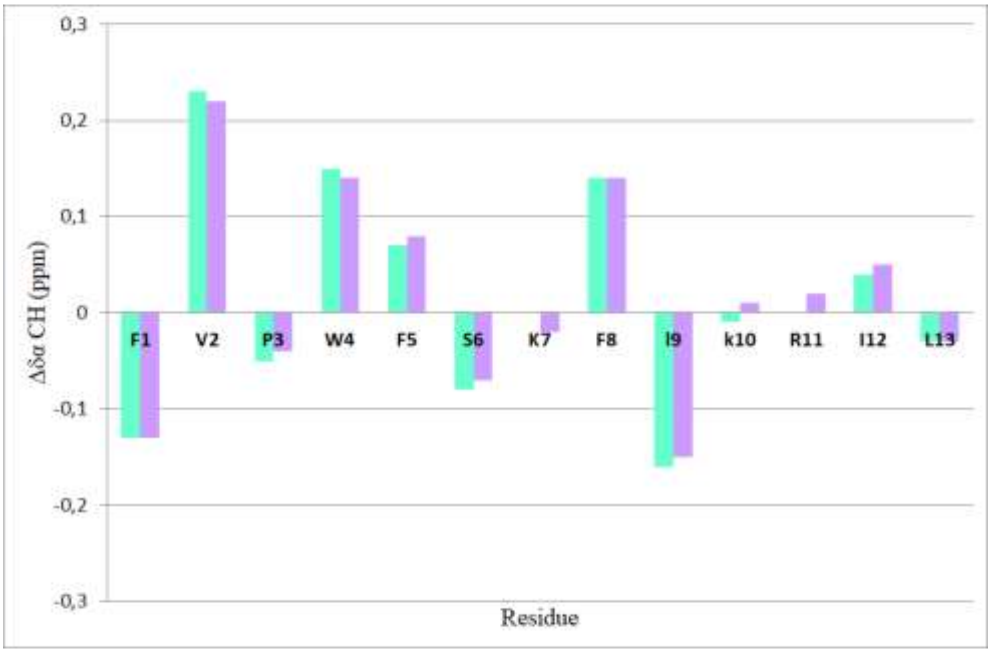

Figure S1. Chemical shift deviations from random coil values of αCH protons of **Pent-1B** (pale cyan bars) and **Dec-1B** (violet bars) in H_2O/D_2O (90/10, *v/v)*.

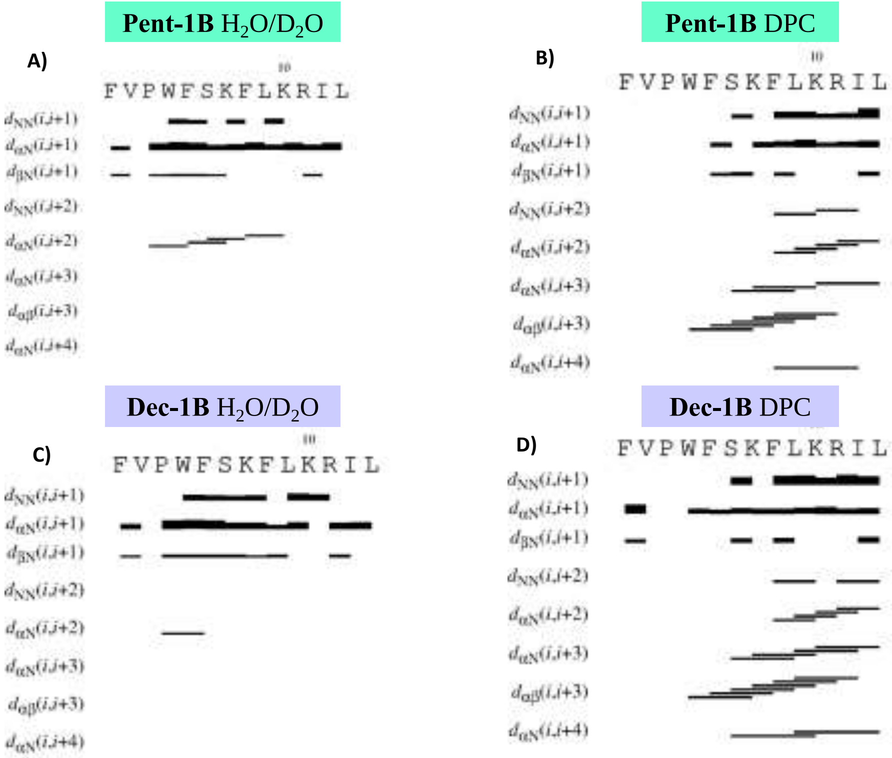

Figure S2. Most relevant NOE contacts of peptides in different media: A) **Pent-1B** H_2O/D_2O 90/10 v/v; B) **Pent-1B** in DPC 20 mM, DPC/peptide ratio R: 67; C) **Dec-1B** H_2O/D_2O 90/10 v/v; D) **Dec-1B** in DPC 40 mM, DPC/peptide ratio R: 67.

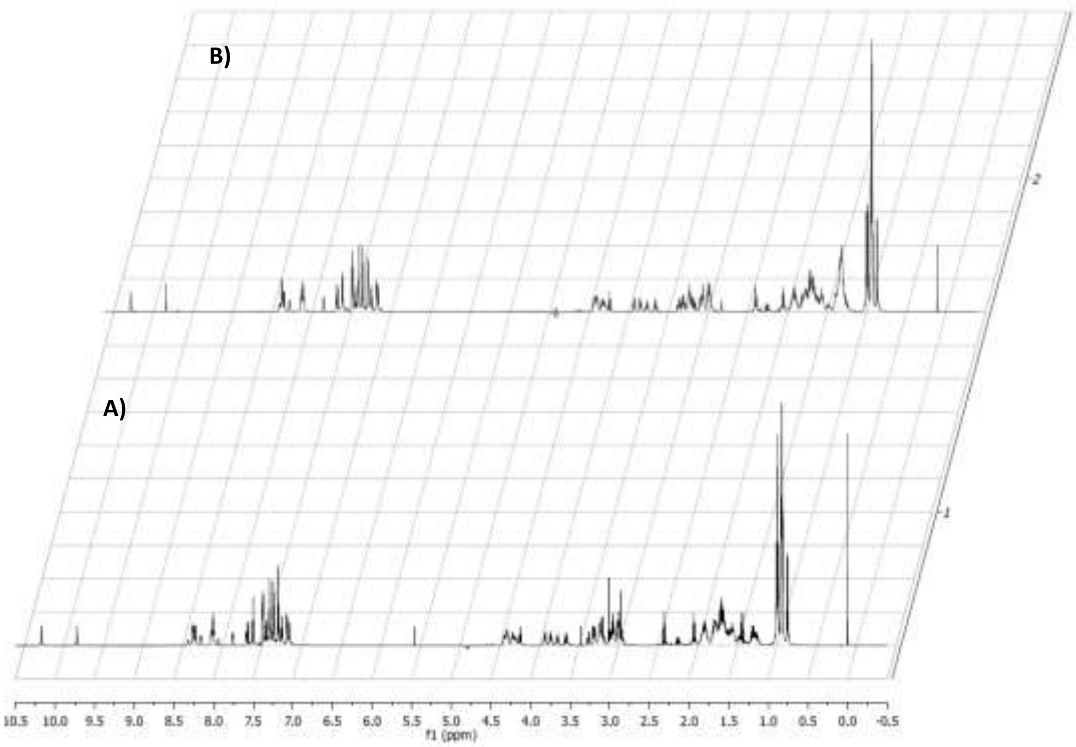

Figure S3. 700 MHz 1D 1H spectra of A) **Pent-1B** (black) and B) **Dec-1B** (dark grey) in H_2O/D_2O (90:10, *v:v*)

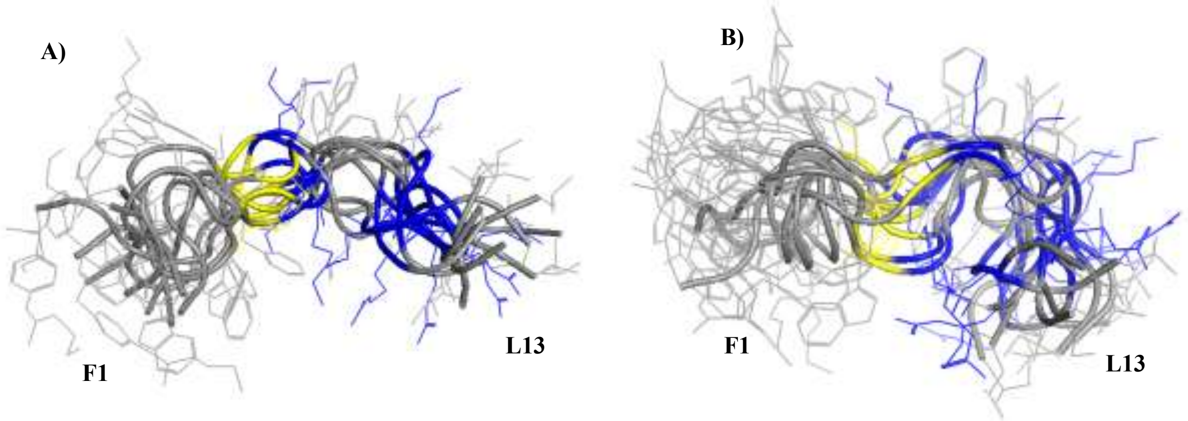

Figure S4. Cartoon representation of the best 10 NMR/CYANA structures in H_2O/D_2O (90/10, *v:v*) of A) **Pent-1B** and B) **Dec-1B**, visualized by PYMOL. Side-chains are shown as sticks coloured by amino acid type: grey, hydrophobic; blue, basic; yellow, polar.

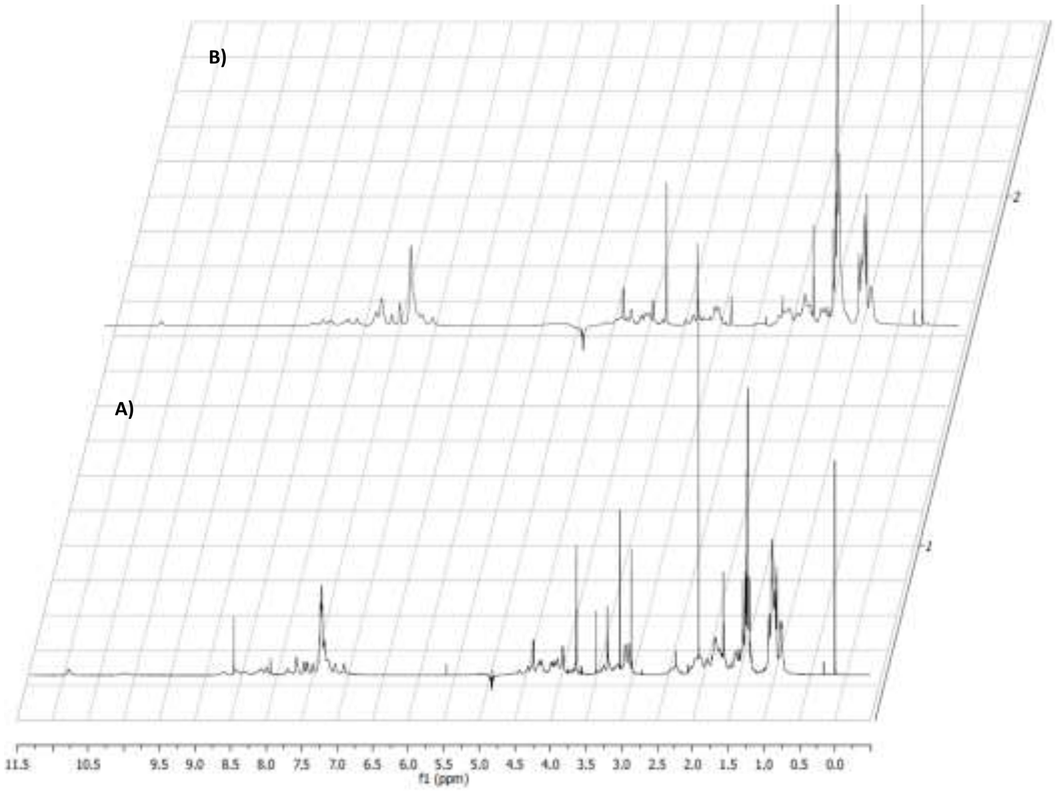

Figure S5. 700 MHz 1D 1H spectra of A) **Pent-1B** (black) and B) **Dec-1B** (dark grey) in DPC, DPC/peptide molar ratio equal to 67.

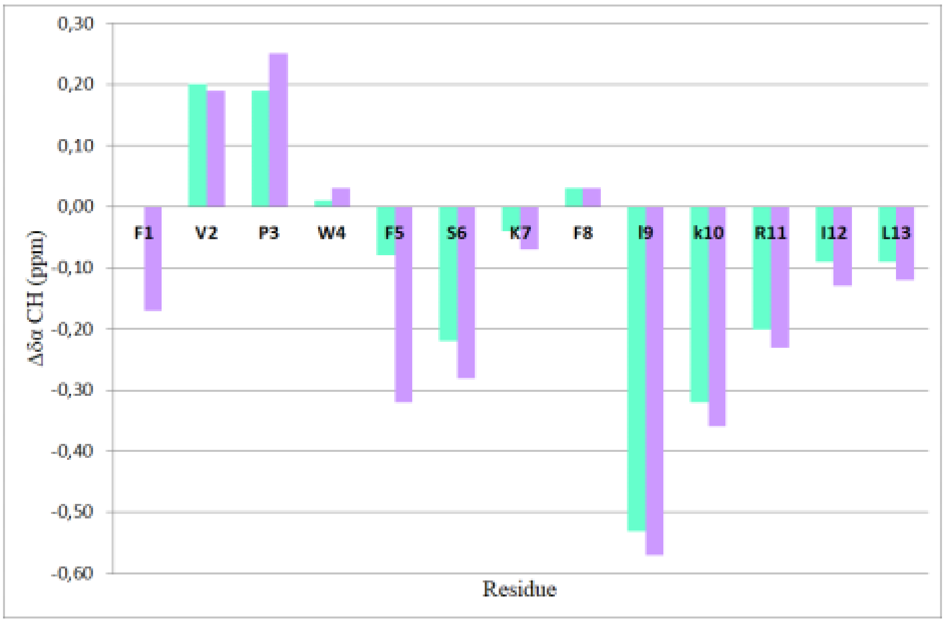

Figure S6. Chemical shift deviations from random coil values of αCH protons of **Pent-1B** in DPC 20 mM, DPC/peptide ratio R: 67 (pale cyan bars) and **Dec-1B** in DPC 40 mM, DPC/peptide ratio R: 67 (violet bars).
